# UIS2: A Unique Phosphatase Required for the Development of *Plasmodium* Liver Stages

**DOI:** 10.1371/journal.ppat.1005370

**Published:** 2016-01-06

**Authors:** Min Zhang, Satish Mishra, Ramanavelan Sakthivel, Beatriz M. A. Fontoura, Victor Nussenzweig

**Affiliations:** 1 Department of Pathology, New York University School of Medicine, New York, New York, United States of America; 2 HIV and Malaria Vaccine Program, Aaron Diamond AIDS Research Center, Affiliate of The Rockefeller University, New York, New York, United States of America; 3 Division of Parasitology, CSIR-Central Drug Research Institute, Lucknow, Uttar Pradesh, India; 4 Department of Cell Biology, University of Texas Southwestern Medical Center, Dallas, Texas, United States of America; Monash University, UNITED KINGDOM

## Abstract

*Plasmodium* salivary sporozoites are the infectious form of the malaria parasite and are dormant inside salivary glands of *Anopheles* mosquitoes. During dormancy, protein translation is inhibited by the kinase UIS1 that phosphorylates serine 59 in the eukaryotic initiation factor 2α (eIF2α). De-phosphorylation of eIF2α-P is required for the transformation of sporozoites into the liver stage. In mammalian cells, the de-phosphorylation of eIF2α-P is mediated by the protein phosphatase 1 (PP1). Using a series of genetically knockout parasites we showed that in malaria sporozoites, contrary to mammalian cells, the eIF2α-P phosphatase is a member of the PP2C/PPM phosphatase family termed UIS2. We found that eIF2α was highly phosphorylated in *uis2* conditional knockout sporozoites. These mutant sporozoites maintained the crescent shape after delivery into mammalian host and lost their infectivity. Both *uis1* and *uis2* were highly transcribed in the salivary gland sporozoites but *uis2* expression was inhibited by the Pumilio protein Puf2. The repression of *uis2* expression was alleviated when sporozoites developed into liver stage. While most eukaryotic phosphatases interact transiently with their substrates, UIS2 stably bound to phosphorylated eIF2α, raising the possibility that high-throughput searches may identify chemicals that disrupt this interaction and prevent malaria infection.

## Author Summary

Malaria is transmitted to humans by female mosquitoes as they take a blood meal. *Plasmodium* sporozoites are the infectious and quiescent forms of malaria parasites, which reside in the salivary glands of mosquitoes. Global protein synthesis is inhibited in sporozoites through phosphorylation of the translational factor eIF2α. However, the development of the parasites in the host liver requires de-phosphorylation of eIF2α-P. We find that a unique *Plasmodium* phosphatase termed UIS2 de-phosphorylates eIF2α-P in malaria. The eIF2α is highly phosphorylated in the *uis2* mutant sporozoites. The *uis2* mutant parasites did not change their morphology after delivery into the host and could not properly infect the host. We also showed that UIS2 expression was inhibited by the Pumilio protein Puf2. However, this repression was relieved when sporozoites developed into liver stage. In sum, our findings revealed a new mechanism that evolved to control eIF2α dephosphorylation and suggest that identification of UIS2 inhibitors may be useful in anti-malaria therapy.

## Introduction

Malaria is a mosquito-borne infectious disease of humans and other animals caused by parasitic protozoans of the genus *Plasmodium*. In 2013, there were 198 million cases of malaria and 584,000 fatalities (WHO world malaria report 2014), underscoring its role as a major pathogen. Sporozoites are the infectious and quiescent forms of the malaria parasite residing in the salivary glands of *Anopheles* mosquitoes. Malaria transmission begins with the injection of salivary sporozoites (Ssp) into the skin of a vertebrate host by infected mosquitoes. The parasites enter the blood circulation and rapidly invade hepatocytes where the crescent-shaped sporozoite progressively transforms into a spherical liver stage (or exo-erythrocytic stage, EEF). Many genes required for the Ssp transformation into liver stages are transcribed in the Ssp [1–3]. However, translation is repressed by phosphorylation of eIF2α by eIK2 kinase, also named UIS1 (UIS, Upregulated in Infective Sporozoites) [4, 5]. If the *eIK2* kinase *uis1* is knocked out, the Ssp transcripts are translated prematurely while sporozoites are still inside the salivary glands of mosquitoes [4]. During the normal parasite cycle, liver-stage transcripts are only translated when Ssp enter hepatocytes and eIF2α-P is de-phosphorylated [6]. Thus, Ssp quiescence is regulated by phosphorylation and de-phosphorylation of eIF2α.

Parasites rapidly multiply inside hepatocytes and generate thousands of merozoites that enter the blood and infect host erythrocytes where they grow, multiply, and transform into schizonts that contain additional infective merozoites. Following entry of merozoites into erythrocytes, a phosphatase must de-phosphorylate eIF2α-P to permit the completion of the parasite’s cycle [7]. Treatment of Ssp with salubrinal, a specific inhibitor of eIF2α phosphatase [8], markedly increases eIF2α phosphorylation in the parasite and inhibits their transformation into liver stages [4, 6]. The central role of eIF2α-P phosphatases in the *Plasmodium* life cycle is highlighted by the observation that the parasites are not viable if they bear the phosphomimetic mutation Ser59Asp in eIF2α that cannot be de-phosphorylated [7]. Nevertheless, no eIF2α-P phosphatase has been identified in Ssp.

In mammalian cells, the de-phosphorylation of eIF2α-P is mediated by the PP1 phosphatase whose activity requires the co-factor GADD34 (Growth Arrest and DNA Damage-Inducible Protein) or its homologue CReP [9]. The substrate specificity of PP1 and its localization are regulated by association with these co-factors [10]. Nevertheless, GADD34/CReP is absent in *Plasmodium* and the molecular mechanism of eIF2α-P de-phosphorylation in the parasite is still unknown. Here we show that the knockout of *pp1* in *P*. *berghei* sporozoites did not affect Ssp quiescence or the levels of eIF2α phosphorylation. These findings excluded a central role of PP1 in the transformation of Ssp into liver stages. We provide evidence that the eIF2α-P phosphatase in *Plasmodium* is a unique serine/threonine phosphatase belonging to the PP2C/PPM family and termed UIS2 [11]. We also show that expression of this phosphatase is regulated at the protein level to support proper parasite development.

## Results

### PP1 is not the eIF-2α-P phosphatase in *Plasmodium* Ssp

**Fig 1:**
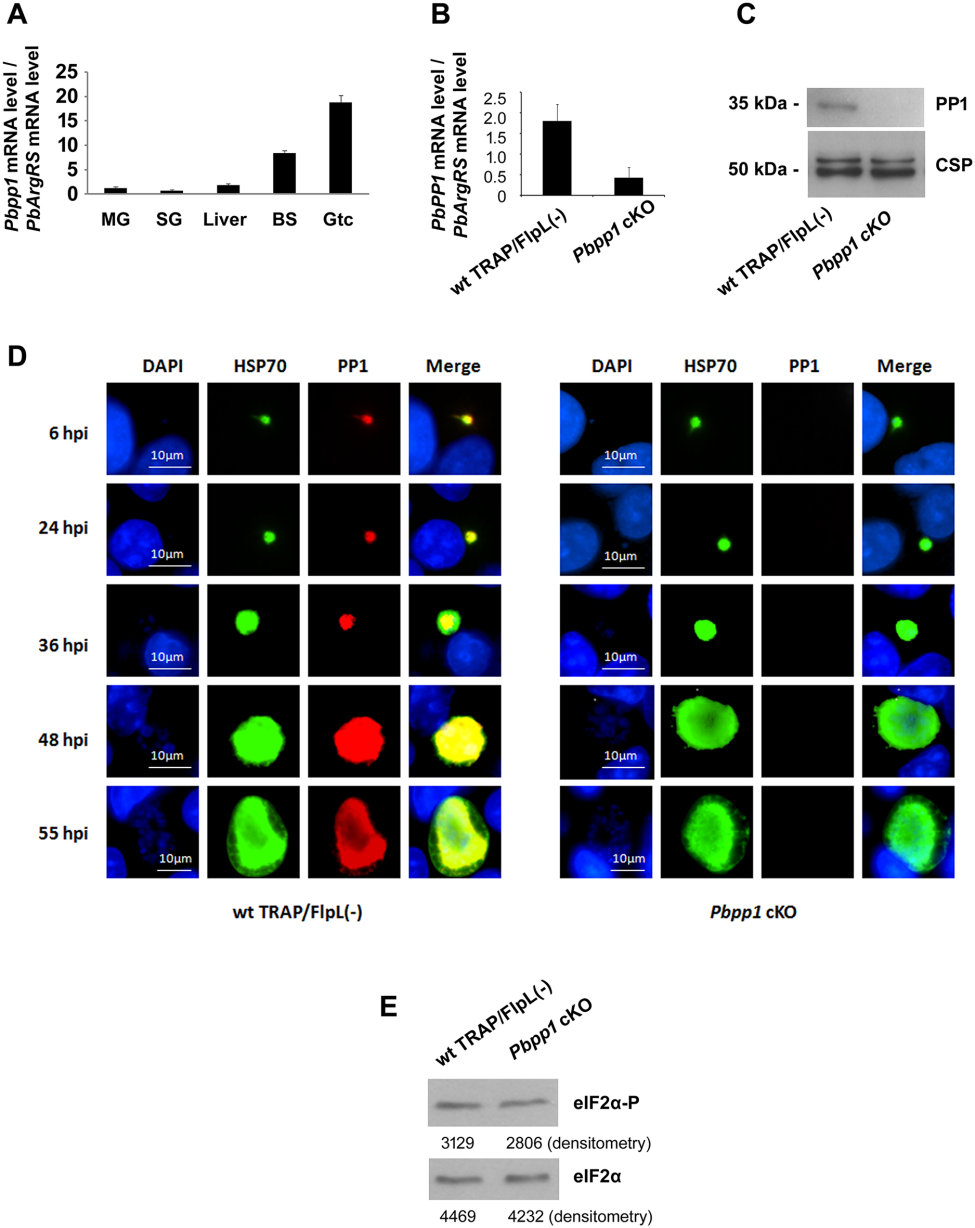
Characterization of *Plasmodium pp1* (*PBANKA_102830*). *(A) pp1* mRNA levels during the *P*. *berghei* life cycle. *Pbpp1* mRNA levels were analyzed by real-time PCR using cDNAs from the different stages of *P*. *berghei*. The *arginyl-tRNA synthetase* (*PbArgRS*, *PB000094*.*03*.*0*) was used as internal control. Each value is the mean ± SD of two independent experiments. MG: midgut; SG: salivary gland; Liver: liver stages; BS: asexual blood stages; Gtc: gametocyte. *(B) Pbpp1* mRNA levels in *Pbpp1* cKO Ssp were quantified by qPCR. P = 0.041. P value was calculated by t test. Shown are mean ± SD of two independent experiments. *(C)* Immunoblot analysis of *Pbpp1* cKO and wt TRAP/FlpL(-) Ssp using anti-PbPP1 serum. CSP was used as control. *(D)* The liver stage development of *Pbpp1* cKO Ssp in HepG2 cells was indistinguishable from the wild type TRAP/FlpL(-) Ssp. Hepatic parasites were stained with anti-PbHSP70 and anti-PbPP1 antibodies at 6h, 24h, 36h, 48h, and 55h post-infection. Bar, 10 μm. *(E)* eIF2α phosphorylation level in *Pbpp1* cKO sporozoites was indistinguishable from wild type. Five hundred thousand wt TRAP/FlpL(-) or *Pbpp1* cKO sporozoites were dissected from mosquito salivary glands. Levels of PbeIF2α-P and total PbeIF2α were quantified by densitometry analysis of immunoblots performed with antibodies against anti-eIF2α-P and anti-total eIF2α [15, 16]. Values are shown below the bands. Results were similar in two independent experiments.

Plasmodium *pp1* transcription takes place during the erythrocytic cycle (Fig 1A), which occurs predominantly in gametocytes. To test whether PP1 phosphorylates eIF-2α, we attempted to knock out *pp1* in *P*. *berghei* erythrocytic schizonts (S1 Fig and S1 Table) but two attempts failed, supporting previous findings demonstrating that this gene is essential for blood stage development [12, 13]. In fact, we showed that high levels of *pp1* mRNA was present in the erythrocytic cycle (Fig 1A). We obtained instead a *P*. *berghei pp1* conditional knockout (cKO) using the FlpL/FRT site-specific recombination system (S2 Fig) [14]. In these *Pbpp1* cKO parasites, *pp1* locus was intact in the blood stages (S2 and S3 Figs) and *pp1* genomic locus was disrupted only when the parasites were developing into sporozoites in mosquito (S3B Fig) [14]. The *pp1* mRNA level was significantly decreased in these mutants (Fig 1B) and PP1 protein was not detected in *Pbpp1* cKO Ssp (Fig 1C). The *pp1* cKO parasites completed the life cycle in *Anopheles* mosquitoes and produced similar numbers of midgut and salivary gland sporozoites as the wild type (wt) (S4 Fig). This is consistent with the low level of *Pbpp1* mRNA in the mosquito vector (Fig 1A). The mutant sporozoites then developed into liver stages and produced hepatic merozoites *in vitro* as shown by IFA (Fig 1D). PP1 was disrupted in 96.3% (SD +/- 4.6%) of the hepatic schizonts (S5 Fig). Quantitative PCR analysis showed that the liver stage development of *Pbpp1* cKO Ssp in HepG2 cells and in mice was indistinguishable from the wild type TRAP/FlpL(-) Ssp (S6 Fig). However, the *pp1* cKO merozoites exiting the hepatocytes did not infect the mouse blood (S7 Fig). Thus, these findings corroborate the essential role of *pp1* in the parasite´s erythrocytic cycle. In addition, the levels of phosphorylation of eIF2α in wt and *pp1* cKO sporozoites were indistinguishable by immunoblot (Fig 1E). In sum, PP1 is not the enzyme that de-phosphorylates eIF2α-P, which is required for liver stage transformation of Ssp.

### UIS2 Interacts with eIF2α-P

**Fig 2:**
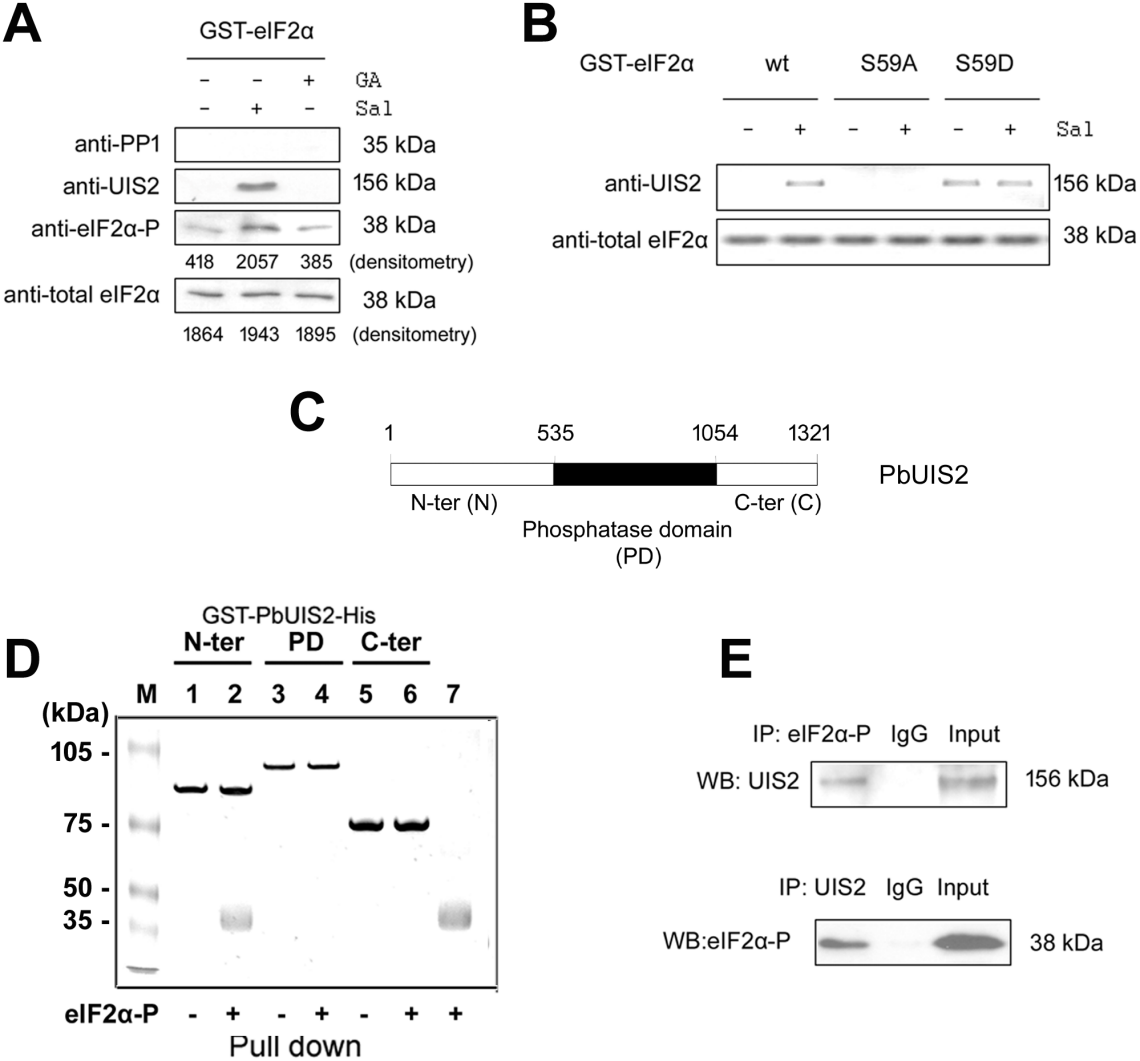
The N-terminus of UIS2 binds eIF2α-P. (A) PfeIF2α-P interacts with endogenous UIS2. In these experiments we used the codon-optimized eIF2α of *P*. *falciparum* [7] that shares 89% identity with its *P*. *berghei* ortholog. The immobilized GST-PfeIF2α was incubated with the lysates of *P*. *berghei* blood stage parasites in the presence or absence of Sal (50 μM) or GA (70 μM). The bound proteins were detected by immunoblot using antibodies against PP1, UIS2, phosphorylated eIF2α, and total eIF2α. Levels of PbeIF2α-P and total PbeIF2α were quantified by densitometry analysis. See also S8 Fig: The mouse anti-PbPP1 antibody recognized the 35 kDa endogenous PP1 from the parasite lysates. (B) PfeIF2α*S59D* mutant protein interaction with UIS2. The immobilized GST-PfeIF2α wt, *S59A*, or *S59D* were incubated with the lysates of *P*. *berghei* blood stage parasites, with or without Sal, and UIS2 was detected by immunoblot. (C) Schematic representation of the PbUIS2 coding sequence. UIS2 contains a putative conserved phosphatase domain (PD, 535–1054 amino acids) and *Plasmodium* specific sequences at N- and C- terminus. (D) The N-terminus of PbUIS2 bound PfeIF2α-P. The PbUIS2 N-ter, PD, and C-ter were fused to GST-tag at their N-terminus and His-tag at their C-terminus, respectively. After 2-step affinity purification, the *E*. *coli* expressed fusion proteins were immobilized on glutathione sepharose 4B. After incubation with purified recombinant PfeIF2α-P, the sepharose was washed three times with high-salt NETN buffer (300 mM NaCl, 20 mM Tris-HCl, pH 8.0, 0.5 mM EDTA, and 0.5% (v/v) Nonidet P-40). The retained proteins were detected by SDS-PAGE followed by coomassie brilliant blue staining. Lane 1,3,5: GST and His-tagged PbUIS2 N-ter, PD, and C-ter, respectively. Lane 2,4,6: proteins retained on the glutathione sepharose 4B after pull down assays with PfeIF2α-P. Lane 7, PfeIF2α-P control. The pulled down 38 kDa protein in lane 2 was analyzed by mass spectrometry and identified as PfeIF2α-P. See also S9 Fig. (E) Co-immunoprecipitation (IP) of endogenous complex between UIS2 and eIF2α-P. Whole cell extracts from *P*. *berghei* blood stage parasites were subjected to immunoprecipitation with anti-eIF2α-P or anti-UIS2 antibodies followed by immunoblot analysis.

*Plasmodium* sporozoites up-regulate a unique subset of genes in the mosquito salivary glands collectively termed UIS [5, 17]. UIS1 (eIK2) is an eIF2α kinase that controls the latency of Ssp [4] and UIS2 is the only phosphatase among the 30 *uis* genes [5]. Thus, we reasoned that UIS2 could be a candidate to de-phosphorylate eIF2α-P when Ssp transform into liver stages. To test this possibility, we first performed pull-down assays to determine whether UIS2 interacts with eIF2α-P using extracts from *P*. *berghei* blood stage parasites. To inhibit endogenous phosphatases in the extracts, we added either salubrinal [Sal, a selective inhibitor of eIF2α phosphatase [4, 8]], or guanabenz acetate [GA, a selective inhibitor of PP1 [18]]. The lysates were then incubated with immobilized GST-PfeIF2α. The beads were extensively washed and the bound parasite proteins were detected by immunoblot analysis. We found that endogenous UIS2 was pulled-down by GST-eIF2α from the lysates containing Sal but not from those lysates containing GA (Fig 2A top 2 panels and S8 Fig). The pull down of UIS2 was associated with the phosphorylation of GST-PfeIF2α (Fig 2A bottom 2 panels), indicating that UIS2 only bound phosphorylated PfeIF2α. Further evidence for the specificity of UIS2/eIF2α-P interaction was obtained by generating mutants of PfeIF2α in which the regulatory ser59 was substituted either with alanine or with aspartic acid that mimics a phosphorylated serine. The PfeIF2α*S59D* bound UIS2 but PfeIF2α*S59A* did not interact with UIS2 (Fig 2B).

*Plasmodium* UIS2 contains a predicted metallo-phosphatase domain enclosed by large N and C-terminal domains (Fig 2C). To determine which domain binds PfeIF2α-P, we expressed the recombinant domains of UIS2 in *E*. *coli*. We show in Fig 2D that only the UIS2 N-terminus stably bound to eIF2α-P but not to non-phosphorylated PfeIF2α (S9 Fig). The association between UIS2 and eIF2α-P under physiological conditions was documented in extracts of *P*. *berghei* erythrocytic parasites by co-immunoprecipitation with anti-eIF2α-P or anti-UIS2 antibodies followed by immunoblot analysis (Fig 2E).

### Properties of the UIS2 phosphatase domain

**Fig 3:**
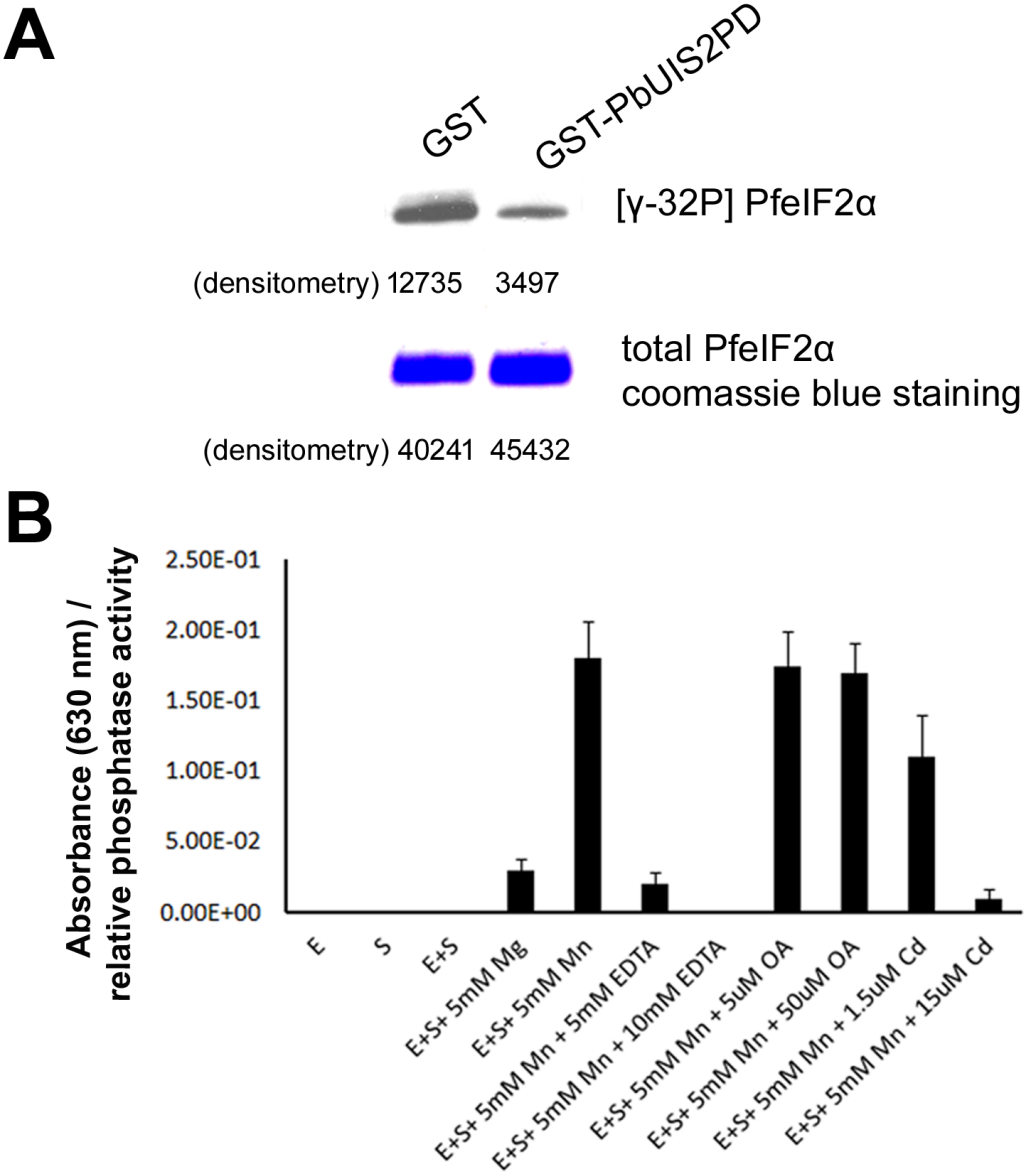
PbeIF2α-P phosphatase activity of UIS2. (A) PbUIS2 phosphatase domain (PD) dephosphorylated eIF2α-P i*n vitro*. PfeIF2α-P[γ-^32^P] was incubated with GST-PbUIS2PD in the presence of 5mM MnCl_2_. (B) Characterization of PbUIS2 as a PP2C/PPM phosphatase. PbUIS2PD phosphatase activity was measured by malachite green colorimetric assay in the presence of EDTA, okadaic acid (OA), or CdCl_2_. E: GST-PbUIS2PD; S: eIF2α-P. The inorganic phosphate was detected with malachite green by measuring the absorbance at 630 nm. Each value is the mean ± SD of three independent experiments.

Next, we tested whether the UIS2 phophatase domain dephosphorylated eIF2α-P. Indeed, we found that the recombinant GST-PbUIS2PD was able to dephosphorylate eIF2α-P *in vitro* (Fig 3A). The activity of the phosphatase was inhibited by EDTA and Cd^2+^ (Fig 3B), which are inhibitors of the PP2C/PPM [19, 20], but was unaffected by okadaic acid (Fig 3B), which inhibits PP1 (IC50 = 15–20 nM) and PP2A (IC50 = 0.1 nM). The phosphatase displayed a strong preference for Mn^2+^ over Mg^2+^ (Fig 3B). Thus, PbUIS2 activity is similar to that of the PP2C/PPM family of phosphatases [21].

### Generation of *uis2* cKO parasites

**Fig 4:**
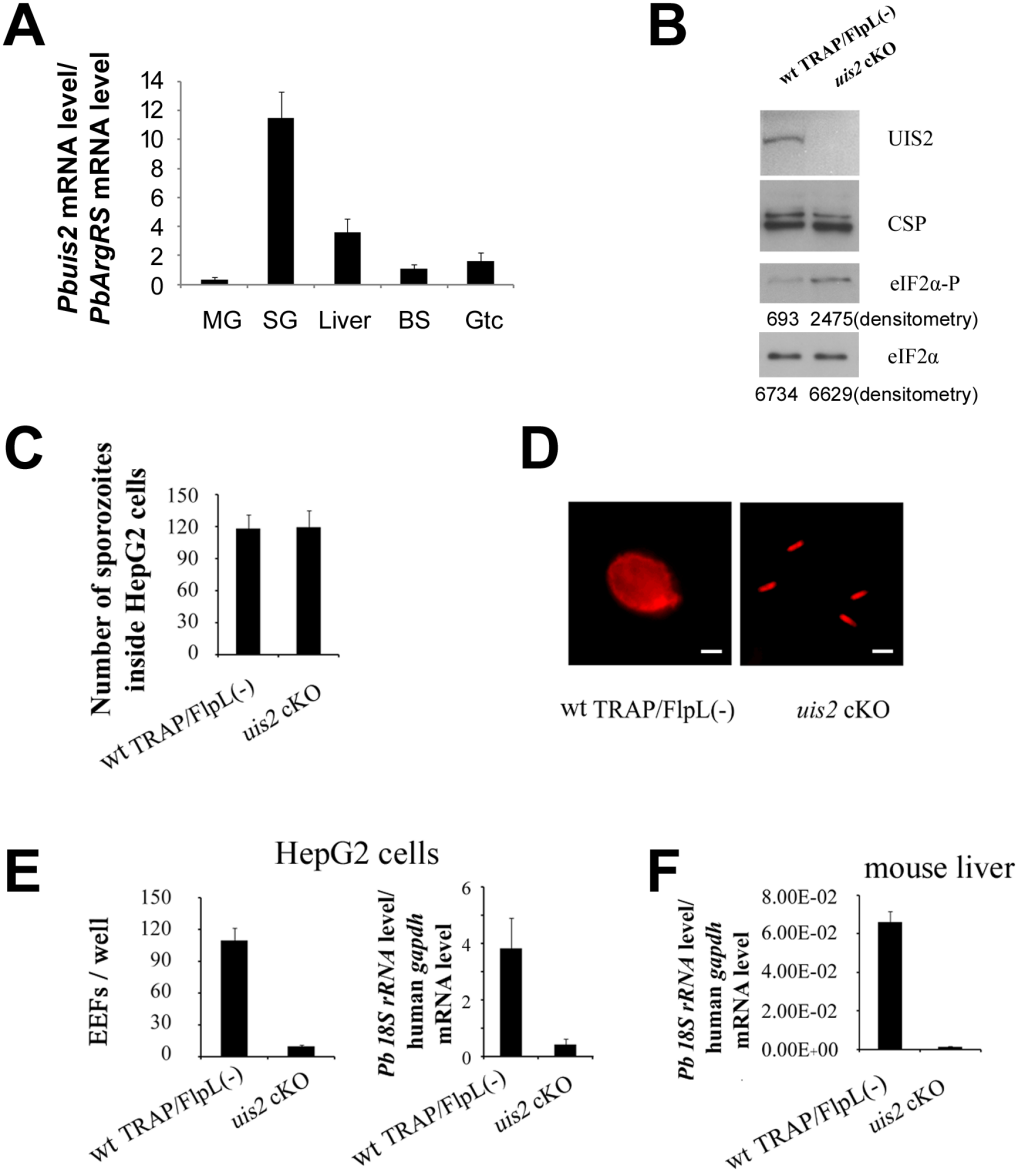
Phenotypes of *uis2 cKO parasites*. (A) mRNA levels of *uis2* in the *P*. *bergthei* life cycle. *Pbuis2* (*PBANKA_132800*) mRNA levels were analyzed by real-time PCR using cDNAs from different stages of *P*. *berghei*. mRNA level was normalized to arginyl-tRNA synthetase (*PbArgRS*, PB000094.03.0). MG: midgut; SG: salivary gland; Liver: liver stages; BS: asexual blood stages; Gtc: gametocytes. Each value is the mean ± SD of two independent experiments. (B) Phosphorylation level of eIF2α was higher in *uis2* cKO Ssp. Levels of UIS2, PbeIF2α-P and total PbeIF2α from 5X10^5^ Ssp are shown by immunoblots. CSP was used as a control. Values of densitometry analysis are shown below the bands. This experiment was repeated three times and similar results were obtained. (C) The mutant sporozoites invaded HepG2 cells as effectively as wt parasites. Wild-type TRAP/FlpL(-) and *uis2* cKO Ssp were added to HepG2 cells and fixed 1 h post-infection. The parasites inside and outside the HepG2 cells were quantified by the hepatocyte invasion assay. Each value is the mean ± SD of two independent experiments. *(* ***D*** *)* The mutant parasites maintained the crescent shape in contrast to the round shape of the wt parasites in HepG2 cells. Wt TRAP/FlpL(-) and *uis2* cKO Ssp were added to HepG2 cells and were detected 48 h post-infection by immunofluorescence using antibodies against the liver stage antigen UIS4. Bars, 10 μm. (E) The development of *uis2* cKO sporozoites was blocked inside HepG2 cells. *P*. *berghei* Ssp infectivity of HepG2 cells was evaluated 48 h post-infection by counting exo-erythrocytic stage (EEF) numbers and measuring18S rRNA level. The left panel shows the mean of EEF numbers ± SD of two independent experiments. The right panel shows liver-stage parasite burden measured by real-time RT-PCR. (F) The development of *uis2* cKO sporozoites was blocked in the mouse liver. C57BL/6 mice (five per group) were injected intravenously with 1×10^4^ wt TRAP/FlpL(-) or with the same number of *uis2* cKO Ssp. Liver-stage parasite burden was measured 42 hours post-infection by real-time RT-PCR. Each value is the mean ± SD of two independent experiments.

We then investigated the expression pattern and levels of the phosphatase *uis2* during the *P*. *berghei* life cycle (Fig 4A). Transcripts were detected during the liver, erythrocytic and mosquito stages. Notably, *uis2* mRNA level increased substantially when midgut sporozoites entered the salivary glands of *Anopheles*. This is consistent with the results of subtractive cDNA hybridization between *P*. *berghei* Ssp and midgut sporozoites [5] and with the comparative microarray analysis of *P*. *yoelii* midgut sporozoites and Ssp [17, 22]. The presence of the UIS2 protein in the Ssp was then detected by immunoblot (Fig 4B). The *uis2* transcript is also present in the blood stages (Fig 4A). The presence of UIS2 in the *P*. *berghei* blood stage parasites was detected by pull down and co-IP assays (Fig 2). To further investigate the *uis2* function, we first tried to knock out the gene in *P*. *berghei* blood stages where *uis2* mRNA levels are very low (Fig 4A); however, several attempts failed (S10 Fig). This result indicated that *uis2* is essential for the development of the *Plasmodium* erythrocytic cycle. Next, we utilized the yeast FlpL/FRT site-specific recombination system to generate the cKO of the *uis2* gene (S1 Text and S11A Fig) [14]. In the *uis2* cKO construct, the 3’UTR of *TRAP* together with DHFR flanked by 2 FRT sites were inserted after the stop codon of *uis2*. We then demonstrated the correct integration of the *uis2* cKO clone in the *uis2* genome locus (S11B Fig). The *FlpL* recombinase was under the control of the *TRAP* gene promoter that is only transcribed when the parasite reaches the mosquito midgut. Since there was no expression of FlpL recombinase in the blood stages, the *uis2* cassette was not affected. When *uis2* cKO reached the mosquito midgut, the *uis2* locus was disrupted (S11C Fig). The disruption of *uis2* expression was confirmed by immunoblot with specific antibodies (Fig 4B).

### UIS2 is essential for Plasmodium development in liver stages

The *uis2* cKO parasite developed normally in the mosquito vector. The number of Ssp in *uis2* cKO and wt parasites was very similar (S12 Fig). Next, we compared the phosphorylation levels of eIF2α in Ssp obtained from *uis2* cKO and from wt parasites by immunoblot using specific antibodies [7, 15, 16, 23]. We found that phosphorylation of eIF2α was greatly enhanced in the absence of the *uis2* phosphatase (Fig 4B) and the mutant sporozoites invaded HepG2 cells as effectively as wt (Fig 4C). Nevertheless, the development of the *uis2* cKO Ssp in HepG2 cells was profoundly inhibited two days post-infection: the mutants maintained the sporozoite shape while the wt parasites rounded up and developed into spherical liver stages (Fig 4D). In addition, the number of exo-erythrocytic forms (EEFs) and the *P*. *berghei* 18S rRNA of the *uis2* cKO parasites were profoundly decreased in the liver stages as compared to the wt parasites (Fig 4E and 4F).

When the *uis2* cKO sporozoites were injected into mice either by mosquito bite or by intravenous injection, only 4 out of 11 mice were infected as detected by Giemsa staining of blood smears. In addition, the pre-patent day was delayed for 3 days in the 4 mice infected with the *uis2* cKO sporozoites as compared to wt (Table 1). We then examined the genotype of the blood and liver stage parasites originated by *uis2* cKO sporozoites. The parasites were *uis2* (+) and expressed the UIS2 antigen as the wt (S13 Fig), indicating that the *uis2* locus had not been completely excised by the recombinase FlpL. The incomplete excision in the FlpL/FRT-mediated conditional mutagenesis system has been previously reported [14]. The overall conclusion of these experiments is that *uis2* is essential for liver stage development.

**Table 1:** Infectivity of wt TRAP/Flp(-) and *uis2* cKO sporozoites in C57BL/6 mice.

Infection	wt TRAP/FlpL(-)		*Uis2* cKO	
	infected / total	PPDs* (no. of mice)	infected / total	PPDs* (no. of mice)
mosquito bite§	6 / 6	4(6)	2 / 6	7(2)
intravenous injection#	5 / 5	4(5)	2 / 5	7(2)

* Pre-patent day (PPD): Number of days after sporozoites inoculation until detection of erythrocytic stages by microscopic examination of blood smears.

^§^10 infected mosquitoes per mouse.

^#^ 10,000 sporozoites per mouse.

### Puf2 controls UIS2 Expression

**Fig 5:**
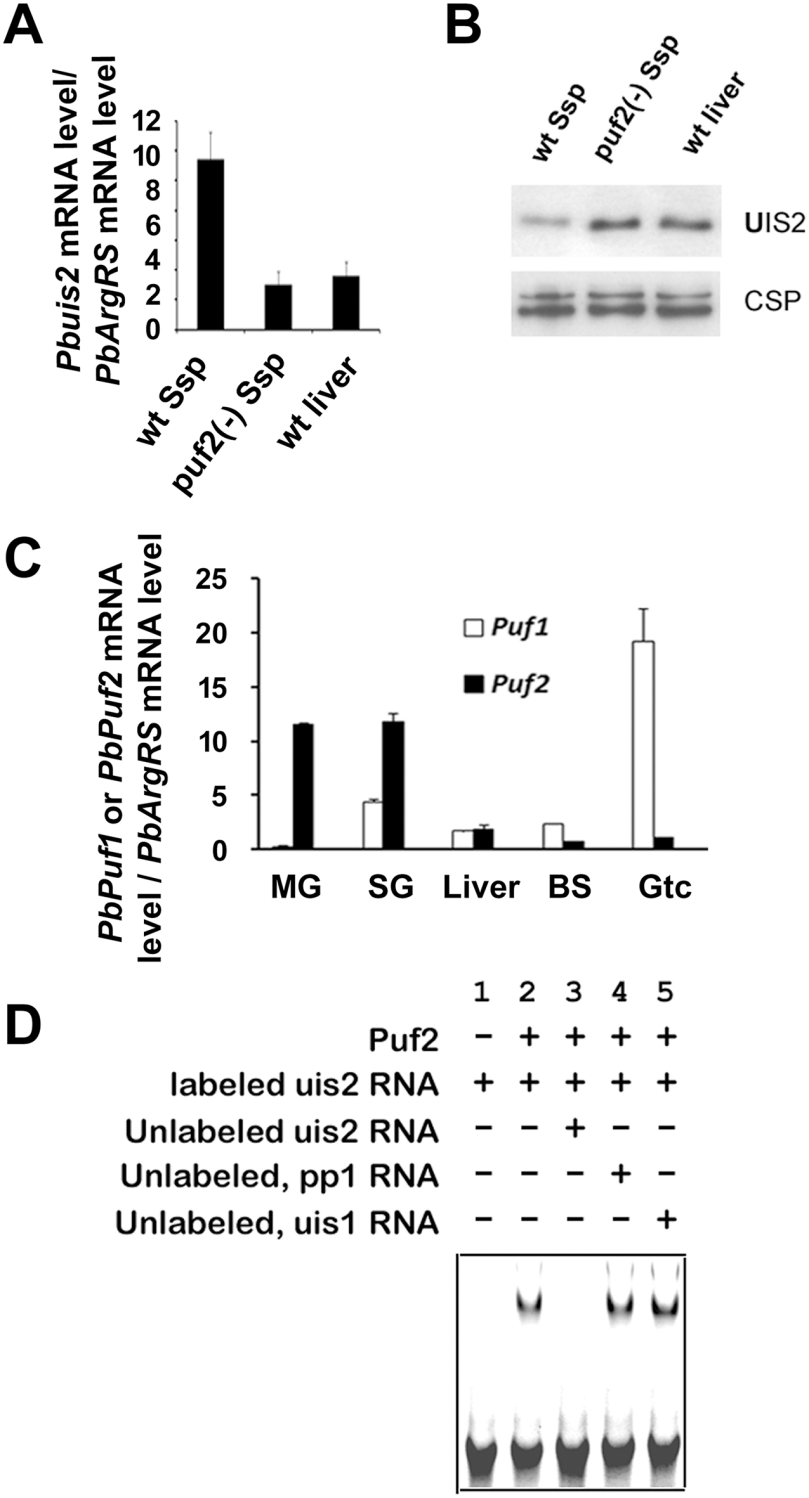
Regulation of UIS2 expression by Puf2 in Ssp. (A) The *uis2* mRNA levels of wt Ssp, *puf2(-)* Ssp, and wt liver stages were measured by Real-time PCR. Each value is the mean ± SD of two independent experiments. (B) Immunoblot depicts UIS2 protein levels in wt Ssp, *puf2(-)* Ssp, and wt liver stages using mouse anti-UIS2 antibodies. CSP was used as internal control (n = 3). (C) mRNA levels of *Puf* proteins at different stages of parasite development. *puf1* and *puf2* mRNA levels were analyzed by real-time PCR using cDNAs from different stages of *P*. *berghei* development. mRNA levels were normalized to the control mRNA, arginyl-tRNA synthetase (PB000094.03.0). MG: midgut; SG: salivary gland; Liver: liver stages; BS: blood stages; Gtc: gametocytes. Each value is the mean ± SD of two independent experiments. (D) The *uis2* mRNA binding to Puf2 was analyzed by RNA gel shift assays. Lane 1: The reaction contains only the biotinylated *uis2*-RNA probe (negative control). Lane 2: The band shift of the *uis2*-RNA probe was detected by the addition of recombinant *P*. *berghei* Puf2 protein. Lane 3: The labeled probe is outcompeted by the addition of a 200-fold excess of un-labeled *uis2*-RNA. Lane 4 and 5: The labeled probe was not outcompeted by the addition of a 200-fold excess of non-specific unlabeled *Pbpp1* or *Pbuis1* probe.

As mentioned above, when midgut sporozoites invade the salivary gland of mosquitoes *uis1* and *uis2* are up-regulated [5]. Yet, the *uis1* kinase is dominant, inhibits protein translation, and maintains the salivary gland sporozoites in a latent state. The eIF2α-P is dephosphorylated when sporozoites are injected into the mammalian host and transform into liver stages [4, 6]. We found that the *uis2* mRNA level was decreased in the liver stages as compared to the sporozoite stage (Fig 5A), which was consistent with the results of previous transcriptome analysis [24]. Nevertheless, *uis2* protein level increased in the liver stages as compared to the sporozoite stages (Fig 5B). These results suggest that *uis2* translation may be repressed in the sporozoite stage and the translational repression may be alleviated in host liver stages. The up-regulation of UIS2 protein level is associated with the eIF2α-P dephosphorylation in host liver [4].

The 3’UTR of *uis2* mRNA contains several putative NREs (Nanos response elements). In *Drosophila* and *C*. *elegans*, Pumilio protein binds to NREs on hunchback mRNA to inhibit its translation [25]. In *Plasmodium* there are two Pumilio proteins (Puf1 and Puf2) [26, 27]. We show in Fig 5C that *puf1* is predominantly transcribed in gametocytes and *puf2* in sporozoites. We then used gel shift assays to document the binding of the Pumilio protein Puf2 to the *uis2* mRNA (Fig 5D). Since *uis2* mRNA level was down-regulated in *puf2(-)* sporozoties (Fig 5A) but the UIS2 protein level was up-regulated (Fig 5B), these results suggest that Puf2 protein may inhibit *uis2* translation in sporozoites. The mRNA and protein levels of UIS2 from the *puf2(-)* sporozoites and wt liver stages were very similar. Taken together, our findings provide the mechanistic explanation to the previous reports showing that *puf2(-)* sporozoites round up and start transforming into liver stages in the mosquito salivary glands [23, 28, 29]. The potential translational repression of *uis2* in Ssp is consistent with the normal development of *Pbuis2* cKO parasites in the mosquito salivary glands where *uis2* is highly transcribed (Figs 4A and S12). As expected, the alleviation of *uis2* repression during liver stages corroborates the defective development of *Pbuis2* cKO parasites during liver stage where *uis2* is significantly translated. Although both *uis* 1 and *uis2* are highly transcribed in Ssp, only *uis1* is dominant. Thus, it is the binding of the Pumilio protein Puf2 to the *uis2* mRNAs that likely inhibits the phosphatase translation. Once translated, US2 binds to eIF2α-P but not to eIF2α. The results imply that small molecules that disrupt the essential UIS2-eIF2α-P interaction will likely interrupt establishment of parasites in hepatocytes and possibly reveal new leads to combat malaria infection.

## Discussion

We show here that UIS2 is the phosphatase that controls the development of the dormant Ssp into the liver stages. Its activity is enhanced by Mn^2+^, inhibited by Cd^2+^, but is not affected by okadaic acid, a powerful inhibitor of PP1 and PP2A phosphatases. Thus, UIS2 belongs to the PP2C/PPM family of phosphatases. The human genome encodes ~ 500 protein kinases, ~2/3 of which are serine/threonine kinases, and approximately 40 serine/threonine phosphatases [30, 31]. In *Plasmodium*, there are ~ 80 protein kinases and ~30 protein phosphatases [13, 32, 33]. These disparate numbers raise the question of how few phosphatases recognize specifically the very large number of phosphorylated proteins. In the case of the PP1 mammalian phosphatase, enzyme specificity and localization are regulated by a large number of multiple co-factors. However, this is not the case for *Plasmodium* UIS2 that encompasses catalytic and regulatory domains within the same polypeptide chain [21]. The N-terminal domain of UIS2 interacts stably with the eIF2α-P substrate placing it in close proximity to the catalytic site. We showed that eIF2α-P interacted with endogenous UIS2 from lysates of blood stage parasites. We did not use lysates from sporozoites because of the repression of *uis2* expression by Puf2. The interaction of UIS2/ eIF2α-P is in sharp contrast to other phosphatases whose interaction with the substrate is unstable and is enhanced by co-factors. In the blood stage, the mRNA level of *uis2* is very low but UIS2 is translated and is essential for the parasite’s blood stage development. However, the function of *uis2* in the blood stage is unknown.

In mammalian cells, PP1 acts in conjunction with the regulatory subunit GADD34 or CReP to de-phosphorylate eIF2α-P. These co-factors are absent in *Plasmodium*. In yeast, the N-terminal extension on eIF2α contains a PP1-binding motif (KKVAF) that enables eIF2α to target PP1 to dephosphorylate eIF2α-P [10, 34]. PP1-binding motif is also absent in *Plasmodium* eIF2α. As shown here, the parasite utilizes instead the PP2C/PPM phosphatase UIS2 to regulate the phosphorylation level of eIF2α. Perhaps additional proteins have evolved to control eIF2α dephosphorylation in organisms that do not contain recognizable homologs of GADD34/CReP or PP1-binding motif (KKVAF) in eIF2α, such as in *Plasmodium*, *S*. *pombe*, and *Aspergillus*.

Small molecules have been useful to distinguish enzyme activities in different organisms. For example, the small molecule GA directly binds to PP1 and selectively inhibits the stress-induced dephosphorylation of eIF2α-P in mammalian cells and in *Toxoplasma* [18, 35]. Nevertheless, GA does not inhibit eIF2α-P de-phosphorylation in *Plasmodium*, supporting our conclusion that PP1 is not the eIF2α-P phosphatase in *Plasmodium*. Instead, Sal is a selective inhibitor of dephosphorylation of eIF2α-P in *Plasmodium* sporozoites [4]. Also shown here, levels of eIF2α-P increased substantially in the Sal treated erythrocytic stages of the parasite. However, in mammalian cells Sal inhibits GADD34/PP1 complex that is responsible for eIF2α dephosphorylation [8], but the molecular mechanism is unknown [36]. There is no ortholog of GADD34, and UIS2 is the eIF2α-P phosphatase. Therefore, the explanation of Sal effect in *Plasmodium* is unknown. Our findings indicate that *Plasmodium* has developed a different strategy to de-phosphorylate eIF2α. Thus, there is a distinct possibility that UIS2 inhibitors will have no side effects on human cells.

**Fig 6:**
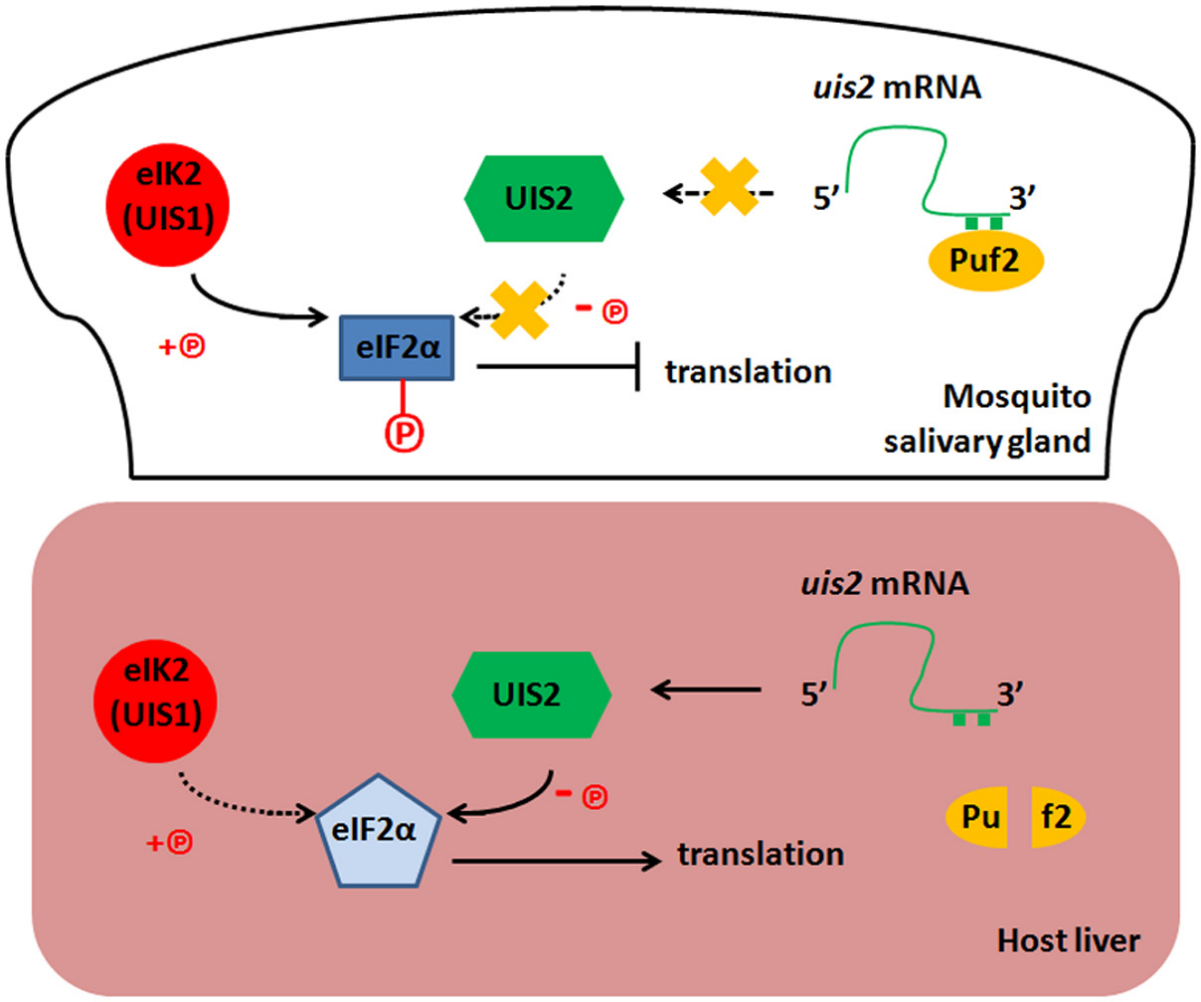
Model for UIS2 function in the *Plasmodium*. The eIF2α kinase eIK2 (also called UIS1) and Pumilio protein Puf2 are highly transcribed and translated in the mosquito salivary gland [3–5, 25, 26]. The highly transcribed eIF2α phosphatase *uis2* mRNA binds to Puf2 and the translation of phosphatase UIS2 is likely repressed. The eIF2α of Ssp is highly phosphorylated since the eIF2α kinase eIK2 activity is dominant, leading to translation inhibition and latency of sporozoites in the mosquito salivary glands. When sporozoites are injected into the mammalian host, the repression of UIS2 translation is probably alleviated, the eIF2α phosphatase UIS2 activity is dominant, eIF2α is dephosphorylated, and Ssp transform into the liver stages. Knockout of *puf2* or *eIK2* contributes to the dominance of UIS2 and the mutant sporozoites inside of mosquito salivary glands prematurely transform into liver stages [4, 23, 28, 29].

We have previously reported that *uis1* encoded the eIF2α kinase eIK2 [4] and here we show that *uis2* encodes the eIF2α phosphatase. It is to be expected that *uis2* (-) and *uis1* (-) Ssp have contrasting phenotypes. The *uis2* (-) Ssp maintained sporozoite shape 48 hours post-invasion into host hepatocytes (Fig 4D). This is in contrast to the *uis1* (-) Ssp, which prematurely transform into spherical liver stages in mosquito salivary glands [4, 23, 28, 29]. UIS2 is highly transcribed and translationally repressed in Ssp, but the repression is alleviated when the dormant Ssp transform into liver stages (Figs 4A, 5A and 5B). The translation of *uis2* is tightly controlled by the Pumilio protein Puf2. In *Drosophila* and *C*. *elegans* Pumilio proteins bind to specific nucleotide motifs at the 3´ UTRs sequences of hunchback mRNAs to inhibit their translation [25]. In *Plasmodium* there are two Pumilio proteins (Puf1 and Puf2) [26]. *Puf1* and *Puf2* are transcribed predominantly in gametocytes and sporozoites, respectively [3, 25, 26]. The function of *Plasmodium* Puf1 is unknown and Puf2 is essential to maintain the infectivity of malaria Ssp [23, 28, 29]. Puf2 likely represses translation of *uis2* mRNA in the mosquito Ssp. In *puf2* (-) parasites, *uis2* mRNA is translated prematurely and the parasites progressively transform into liver stages while they reside in the salivary glands [23, 28, 29]. When wt Ssp parasites are injected into the mammalian host, the translational repression of *uis2* is probably alleviated, eIF2α-P is dephosphorylated [4], and liver stage messages are decoded (Fig 6).

In sum, the conditional knockouts of *pp1* and *uis2* in Ssp together with the biochemical evidence demonstrating that UIS2 binds to and de-phosphorylates eIF2α-P reveal that UIS2, and not PP1, is the eIF2α phosphatase in *Plasmodium* Ssp. These findings raise the possibility of using high-throughput screenings of small molecules to disrupt UIS2-eIF2α-P interaction, which is essential for the parasite’s survival, and perhaps lead to the discovery of new drugs to interrupt the parasite’s development in hepatocytes.

## Materials and Methods

### Ethics statement

All animal work has been conducted according to Institutional Animal Care and Use Committee (IACUC) Laboratory Animal Protocol: 140102.

### Stage-specific mRNA levels of *uis2* and *pp1*

*Pbuis2* (*PBANKA_132800*) and *Pbpp1* (*PBANKA_102830*) mRNA levels were analyzed by real-time RT-PCR using cDNA prepared from blood, liver and mosquito-stage parasites of *P*. *berghei*. Axenic liver stages of the parasite were prepared by incubation of *P*. *berghei* salivary gland sporozoites at 37°C in DMEM plus 10% FBS for 6 hours as described previously [37, 38]. Total RNAs were extracted using TRIzol reagent and treated with DNase. The absence of genomic DNA contamination was confirmed by PCR amplification on same-treated RNA samples that lacked reverse transcription. The specificity of amplification for each PCR product was confirmed by dissociation curve analysis. Real-time PCR was performed using iQ SYBR Green Supermix (Bio-Rad Laboratories), according to the manufacturer’s instructions. The 1ml reaction mix contained 1X iQ SYBR Green Supermix, 300 nM forward/reverse primers, and cDNA reverse-transcribed from 2 μg RNAs. The temperature profile included 95°C for 10 min, 35 cycles of denaturation at 95°C for 15 sec, and annealing/extension at 60°C for 1 min. Transcript expression was normalized to the expression of the control gene, arginyl-tRNA synthetase (*PbArgRS*, *PB000094*.*03*.*0*). The normalized expression was calculated as following: relative amount of *Pbuis2* cDNA / relative amount of *PbArgRS* cDNA. Gene-specific primers were *PbArgRS* (sense 5’- ttggtgattggggaacac-3’, antisense 5’- cttgatataaaagggtcaaac-3’); *Pbuis2* (sense 5’- actgaaaatgaacatgccttacta -3’, antisense 5’- catatgggtgagcttcttcctt-3’); *Pbpp1* (sense 5’-cccgaaaaggaaataaatgg-3’, antisense 5’-ttggagccgaaaataaagtaac-3’).

### Antisera

A fragment of *P*. *berghei* UIS2 (from 413 to 618 amino acids) fused at the C-terminal with GST was expressed in *E*. *coli* and was used to immunize mice. Western blot of *P*. *berghei* sporozoites lysates with the mouse antibody (1:1,000 dilution) revealed a 160 kDa band (endogenous PbUIS2 is 156 kDa). The identity of Puf2 proteins from *P*. *berghei* and *P*. *yoelii* is 95%. The Puf2 antiserum was raised in mice by immunization with the GST-fused *P*. *yoelii* Puf2 recombinant protein. The Puf2 antibody (1:1,000 dilution) recognizes a band of about 60 kDa band in western blots of *P*. *berghei* or *P*. *yoelii* sporozoites lysates. The anti-total eIF2α and anti-eIF2a-P sera were generated in William J. Sullivan Jr.’s lab [15, 16]. The Anti-eIF2a-P sera (1:500 dilution) specifically recognize *Plasmodium* eIF2a-P [4, 7, 23]. Anti-total eIF2α sera (1:1,000 dilution) recognizes both phosphorylated and non-phosphorylated forms of eIF2α from *Toxoplasma* [15] and *Plasmodium* [4, 7, 23].

### eIF2α-P phosphatase activity *in vitro*

The eIF2α-P phosphatase activity was measured by two methods: (1) His-tagged PfeIF2α was incubated with GST-PfPK4 kinase domain and [γ-32P] ATP for 1 hour as described [7] and purified by Ni-NTA affinity chromatography. The [γ-32P] labeled PfeIF2α-P was then used as the phosphatase substrate as following: One μg GST or GSTPbUIS2PD was added into 100 μg [γ-32P] labeled PfeIF2α-P in the assay buffer (20mM Tris-HCl, pH7.0; 50 mM NaCl) with 5 mM MnCl_2_. After 1 hour incubation at 37°C, the total proteins were separated by SDS-PAGE and subjected to autoradiography and coomassie blue staining. (2) Malachite green colorimetric assay: GSTPbUIS2PD in assay buffer (50mM Tris-HCl, pH7.0) was pre-incubated for 30 min in the absence or presence of MgCl_2_, MnCl_2_, CdCl_2_, or okadaic acid. PfeIF2α-P was added and incubated for an additional 30 min. Then, 2 volumes of malachite green reagent (0.15% malachite green, 1% ammonium molybdate and 12.5% concentr*a*ted HCl v/v) were added before reading the absorbance at 630 nm.

### Pull down assay from parasite lysate

*P*. *falciparum* eIF2α coding sequence was codon-optimized and expressed with a GST fusion tag in *E*. *coli* [7]. The GST-PfeIF2a was immobilized and purified on glutathione affinity column. The lysates of *P*. *berghei* blood stage parasites were incubated with immobilized GST-PfeIF2α in the presence or absence of eIF2α phosphatase specific inhibitor Salubrinal (Sal, 50 μM), or PP1 inhibitor Guanabenz acetate (GA, 70 μM) for 2 hours. After 3 times wash with high-salt NETN buffer (300 mM NaCl, 20 mM Tris-HCl, pH 8.0, 0.5 mM EDTA. 0.5% (v/v) Nonidet P-40), the GST-PfeIF2α and its binding proteins were eluted with 10 mM reduced glutathione/ 50 mM Tris-HCL pH 8.0. The eluates were analyzed by immunoblots using antisera specific to PP1, UIS2, phosphorylated eIF2α, and total eIF2α.

### Hepatocyte invasion assay

Ten thousand *P*. *berghei* sporozoites were added to confluent HepG2. After 60 min incubation at 37°C, the HepG2 cells were fixed with 4% paraformaldehyde and blocked with 3% BSA in PBS. To detect the parasites outside the HepG2 cells, the slide was stained with anti-CSP 3D11 Ab [39] followed by Alexa Fluor 594 goat anti-mouse IgG (Molecular Probes). Then, to detect both sporozoites outside and inside hepatocytes [40], the HepG2 cell membranes were permeabilized with 100% chilled methanol. The cells were again blocked and stained with anti-CSP 3D11 Ab followed by Alexa Fluor 488 goat anti-mouse IgG as secondary Ab. Sporozoites were counted under a fluorescent microscope.

### Gel shift assay

GST-tagged PbPuf2 was expressed in *E*. *coli* and purified by affinity chromatography. The biotinylated *uis*2-RNA was synthesized using HiScribe T7 High Yield RNA Synthesis Kit (New England Biolabs). The Puf2 protein was incubated with 5 nM biotinylated *uis2*-RNA in 1X REMSA buffer containing 5% glycerol. Reactions were resolved on a native 6% polyacrylamide gel in 0.5X TBE and transferred to a nylon membrane. Band shifts were detected using Thermo scientific LightShift Chemiluminescent RNA EMSA kit.

## Supporting Information

S1 Text Supporting Method / Parasite transfection. (DOCX)

S1 Table Primers. (DOCX)

S1 Fig *Pbpp1* knockout attempts. A double cross-over knockout strategy was used to knockout *Pbpp1*. hDHFR, human dihydrofolate reductase. Parasites were found in the blood smear of mice 24 hours post-transfection of the linearized construct. The mice were then treated with pyrimethamine. No parasites were observed 14 days post pyrimethamine treatment. Two attempts to knockout *Pbpp1* failed. Related to Fig 1.
(TIF)

S2 Fig Strategy to generate *P*. *berghei pp1* cKO parasite. (A) The targeting plasmid for generating the *Pbpp1* cKO contains the 3’ end (700 bp) of the *pp1* coding sequence (box CDS), a fragment including 0.6 kb of TRAP 3’ regulatory sequence (lollipop) and the hdhfr marker cassette (gray box), the plasmid backbone (thick line), and *pp1* 3’ regulatory sequence (500 bp, lollipop). The linearized plasmid was integrated at the cognate locus into wt TRAP/FlpL(-) NK65 parasite via double crossover recombination, generating the *pp1* cKO clone. (B) *Pbpp1* expression cassette is intact in *Pbpp1* cKO blood stage. Swiss Webster mice (5 per group) were injected intra-peritoneally with 200 μl of blood infected with *Pbpp1*cKO or wt TRAP/FlpL(-) parasites (1% parasitemia). The parasitemia of the recipient mice was checked in Giemsa-stained blood smears. Since the FlpL recombinase is expressed in midgut sporozoites, *Pbpp1* is only disrupted in Ssp. Related to Fig 1.
(TIF)

S3 Fig gDNA PCR from the *Pbpp1* cKO parasites. The primers used for integration-specific PCR analysis are indicated as arrows in S2 Fig. (A) Integration-specific PCR analysis of the *uis2* loci of the wt TRAP/FlpL (-) NK65 and *uis2* cKO blood stage clones. Lanes 1 and 4: primers P5+P6; lanes 2 and 5: primer P5+P2; lanes 3 and 6: primer P4+P6. (B) *Pbpp1* locus is disrupted in *Pbpp1* cKO Ssp. PCR amplification from gDNA of intact *uis2* cKO blood stage parasites or from *pp1* cKO Ssp using primers P5+P2 were performed. Control depicts PCR amplification of the eIF2α coding sequence. Related to Fig 1.
(TIF)

S4 Fig Wt TRAP/FlpL(-) and *Pbpp1*cKO parasites produced similar numbers of midgut and salivary gland sporozoites. Sporozoite numbers were counted in three different mosquito cycles. Related to Fig 1
(TIF)

S5 Fig Immunofluorescence assays of *Pbpp1* cKO hepatic schizonts. (A) Twenty thousand wild type TRAP/FlpL(-) or *Pbpp1* cKO sporozoites were added to 1x10^5^ HepG2 cells. Fourty-eight hours post infection hepatic parasites were stained with anti-PbHSP70 and anti-PbPP1. Bar, 100 μm. (B) The HSP70 stained liver stage parasites were counted. The EEF (Liver stage) numbers were indistinguishable between wild type TRAP/FlpL(-) and *Pbpp1* cKO parasites. P value was calculated by t test. (C) The HSP70 stained and PP1 stained liver stage parasites were counted in *Pbpp1* cKO infected HepG2 cells. The *Pbpp1* disruption rate was calculated from 5 independent experiments. Related to Fig 1.
(TIF)

S6 Fig Quantitative PCR analysis of *Pbpp1* cKO Ssp developed in HepG2 cells and in mice. (A) Twenty thousand wild type TRAP/FlpL(-) or *Pbpp1* cKO sporozoites were added to 1x10^5^ HepG2 cells and grown for 6, 24, 36, 48, and 55 h. *P berghei* 18S rRNA copy number was measured by qPCR. Human *gapdh* was used as internal control. There was no significant difference between wt and *Pbpp1* cKO. (B) C57BL/6 mice (6 weeks old, five mice per group) were intravenously injected with 1x10^4^ wt TRAP/FlpL(-) or *Pbpp1*cKO sporozoites. Liver-stage parasite burden was measured 42 hours post infection by qPCR, and shown are the mean ± SD. *Pbpp1* cKO Ssp developed normally in mice. P value was calculated by t test. Related to Fig 1.
(TIF)

S7 Fig Defective erythrocytic stage development in *Pbpp1*cKO sporozoites. (A) Infectivity of wt TRAP/FlpL(-) and *Pbpp1* cKO sporozoites after intravenous injection of C57BL/6 mice. Genotype of blood stage parasites was analyzed from the *Pbpp1* cKO sporozoites infected mice. The parasites were *pp1* (+); therefore, the *pp1* locus had not been disrupted by the recombinase FlpL. The incomplete excision in the FlpL/FRT-mediated conditional mutagenesis system was previously reported by Combe *et al*. [14]. (B) Genotype of blood stage parasites from the *Pbpp1*cKO sporozoites infected mouse performed by gDNA PCR. Lane 1, intact *Pbpp1* cKO blood stage parasites; lane 2, *Pbpp1* cKO Ssp; lane 3, blood stage parasites from a *Pbpp1* cKO sporozoites infected mouse. Primers P5+P7 (S2 Fig) were used to verify the disruption of *Pbpp1* 3’UTR. Primers P5 and P2 were used to verify the intact *Pbpp1* locus. Related to Fig 1.
(TIF)

S8 Fig Immunoblot analysis of endogenous PP1 from the lysates of *P*. *berghei* blood stage parasites. The proteins from 5X10^5^ *P*. *berghei* blood stage parasites were separated by SDS-PAGE followed by Western blot using naive mouse serum (Lane 1) and mouse anti-PbPP1 antibody (Lane 2), respectively. The mouse anti-PbPP1 antibody recognized the 35 kDa endogenous PP1 from the lysates of *P*. *berghei* blood stage parasites. Related to Fig 2.
(TIF)

S9 Fig The N-terminus of PbUIS2 binds to PfeIF2α-P. (A) The N-terminus of PbUIS2 pulled down PfeIF2α-P. The PbUIS2 N-ter was fused to GST-tag at its N-terminus and His-tag at its C-terminus. After 2-step affinity purification, the *E*. *coli* expressed fusion protein (85 kDa) was immobilized on glutathione sepharose 4B. After incubation with purified recombinant PfeIF2α or PfeIF2α-P, the sepharose was washed three times with high-salt NETN buffer (300 mM NaCl, 20 mM Tris-HCl, pH 8.0, 0.5 mM EDTA, and 0.5% (v/v) Nonidet P-40). The retained proteins were detected by SDS-PAGE followed by coomassie brilliant blue staining. Lane 1: PbUIS2 N-ter. Lane 2: proteins retained on the glutathione sepharose 4B after the pull down assays with non-phosphorylated PfeIF2α. Lane 3: non-phosphorylated PfeIF2α input control. Lane 4: proteins retained on the glutathione sepharose 4B after the pull down assays with PfeIF2α-P. Lane 5, PfeIF2α-P control. (B) Mass spectrometry shows that the protein bound to the PbUIS2 N-ter is indeed PfeIF2α-P. The PbUIS2 N-ter pulled down protein was analyzed by mass spectrometry. Peptides denoted in green are peptides identified in our analysis. Each green line under a peptide denotes the number of times the peptide has been identified in this analysis (spectral counts). A red line below a peptide indicates that the corresponding amino acid carries a modification (*in vivo* or *in vitro*). The modification shown here are: C = Carbamidomethylation (+57 for alkylation of cysteines, that is a result of the sample preparation); M = oxidation (+16 for oxygen addition, common in gel digestion); Q, N = deamidation (+1, usually an *in vitro* modification enhanced by storing samples at high pH. Sample digestion was performed at pH = 8 overnight). Related to Fig 2.
(TIF)

S10 Fig *Pbuis2* knockout attempt. A double cross-over knockout strategy used to attempt a knockout *uis2* in *P*. *berghei*. The *uis2* KO plasmid pBC_uis2KO contains a 500-bp PCR fragment from *uis2* coding sequence (CDS, 800–1300 bp), GFP cassette, DHFR cassette, and a 500 bp PCR fragment from the *uis2* C terminal CDS. DHFR, dihydrofolate reductase. Three independent attempts to generate *Pbuis2* knock-out parasite failed.
(TIF)

S11 Fig Generation of conditional *uis2* cKO using the TRAP/FlpL system. (A) Schematic representations of the *uis2* locus in the wild type TRAP/FlpL(-) NK65 clone and in the *uis2* cKO clone. The *uis2* cKO plasmid contains the 3’ end (800 bp) of the *uis2* coding sequence (box CDS), a fragment including 0.6 kb of TRAP 3’ regulatory sequence (lollipop) and the hdhfr marker cassette (gray box), the plasmid backbone (thick line), and *uis2* 3’ regulatory sequence (770 bp, lollipop). The linearized plasmid integrated at the cognate locus into wt TRAP/FlpL(-) NK65 parasite via double crossover recombination, generating the *uis2* cKO clone. The primers used for integration-specific PCR analysis are indicated as arrows. (B) Integration-specific PCR analysis of the *uis2* loci of the wt TRAP/FlpL (-) NK65 and *uis2* cKO clones. Lanes 1 and 4: primers P1+P3; lanes 2 and 5: primer P1+P2; lanes 3 and 6: primer P3+P4. (C) *Pbuis2* locus is disrupted in *Pbuis2* cKO Ssp. PCR amplification from gDNA of intact *uis2* cKO blood stage parasites (lane 1) or *uis2* cKO Ssp (lane 2) using Primers P1+P2. Controls are shown in lanes 3 and 4: PCR amplification of eIF2α coding sequence from *uis2* cKO blood stage parasites (lane 3) and from *uis2* cKO Ssp (lane 4). Related to Fig 4.
(TIF)

S12 Fig Wild type TRAP/FlpL(-) and *Pbuis2*cKO parasites produced similar numbers of midgut and salivary gland sporozoites. Sporozoite numbers were counted in three different mosquito cycles. Related to Fig 4.
(TIF)

S13 Fig Genotype of the liver and blood stage parasites from *uis2* cKO sporozoites. (A) A few *uis2* (+) parasites still remained from the liver stages originated from *uis2* cKO Ssp. The figure represented one of them stained with anti-UIS2 and anti-UIS4 sera. Bars, 10 μm. (B) The genotype of the blood stage parasites originated by *uis2* cKO sporozoites was *uis2* (+). gDNA PCR amplifications were performed using primers P1 and P2 (S11 Fig) and *uis2* expression cassette from intact *uis2* cKO blood stage parasites (lane 1), *uis2* cKO Ssp (lane 2), and the erythrocytic stages parasites from a mouse infected with *uis2* cKO Ssp (lane 3). eIF2α was used as internal control. Related to Table 1.
(TIF)
