# Supplementary material for: UIS2: A Unique Phosphatase Required for the Development of Plasmodium Liver Stages
Source: PLoS Pathog. 2016 Jan 6;12(1):e1005370. doi: 10.1371/journal.ppat.1005370 (PMC4712141; doi:10.1371/journal.ppat.1005370)
Supplement: S1 Text — (DOCX) [file ppat.1005370.s001.docx]

**S1 Text**

**Supporting Method/ Parasite transfection**

The *P. berghei* TRAP/FlpL cKO system was obtained from Robert Menard’s lab [1]. The TRAP/FlpL(-) NK65 parasite genome is integrated with FlpL (thermolabile variant) recombinase under the *TRAP* promoter, which is highly active in sporozoites of mosquito midgut. This parasite shows similar phenotype as wild type *P. berghei* NK65. Here we called TRAP/FlpL(-) NK65 “wild type”. The *uis2* cKO plasmid contains (1) the last 800 bp of the *uis2* coding sequence immediately followed by the first 16 nucleotides of the TRAP 3’ UTR, (2) a first FRT site, (3) the TRAP 3’ UTR (0.6 kb) starting at position +16 after the TRAP stop codon, (4) the hDHFR cassette, (5) the second FRT site, (6) the plasmid backbone, and (7) 770 bp of *uis2* 3’UTR. Transfection of linearized *uis2* cKO plasmid into wt TRAP/FlpL(-) parasites were carried out using standard methodology [2]. Transfected parasites were selected by pyrimethamine and cloned by limiting dilution and growth in mice. The FlpL is expressed when the parasite develops into sporozoites in the mosquito midgut. The recombinase recognizes the FRT sites from the genome of the conditional knockout parasites and excises the DNA between the two FRT sites.

**Supporting References**

1. Combe A, Giovannini D, Carvalho TG, Spath S, Boisson B, Loussert C, et al. Clonal conditional mutagenesis in malaria parasites. Cell Host Microbe. 2009;5(4):386-96. Epub 2009/04/22. doi: S1931-3128(09)00099-7 [pii]

10.1016/j.chom.2009.03.008. PubMed PMID: 19380117.

2. Janse CJ, Ramesar J, Waters AP. High-efficiency transfection and drug selection of genetically transformed blood stages of the rodent malaria parasite Plasmodium berghei. Nat Protoc. 2006;1(1):346-56. Epub 2007/04/05. doi: nprot.2006.53 [pii]

10.1038/nprot.2006.53. PubMed PMID: 17406255.
