# Supplementary material for: UIS2: A Unique Phosphatase Required for the Development of Plasmodium Liver Stages
Source: PLoS Pathog. 2016 Jan 6;12(1):e1005370. doi: 10.1371/journal.ppat.1005370 (PMC4712141; doi:10.1371/journal.ppat.1005370)
Supplement: S1 Table — (DOCX) [file ppat.1005370.s002.docx]

**S1 Table.** Primers

| Primer | Sequence | Description |
| --- | --- | --- |
| G1 | gaagaatatcacattttgttttata | knockout of *Pbpp1* |
| G2 | gccataataaaagttactatatttt |  |
| G3 | cgacataaaaatacagtgatttta |  |
| G4 | atttgatttaatttttaaggtgcgc |  |
| C1 | gctgaaaaatatgaagataaagaattaaac | knockout of *Pbuis2* |
| C2 | gattttgaaacgatagaaagctcatcg |  |
| C3 | ggctcccaatatgcatatatcaaatc |  |
| C4 | tgcgtgcttgcgctctgattacc |  |
| P1 | gtcatatttctaatatatcatttgatc | Integration-specific PCR analysis of the *Pbpp1*and *uis2* loci |
| P2 | caaacgagtttatgagtttattatttaaag |  |
| P3 | ttttatgtttttgccgtgtaatttgtg |  |
| P4 | gtaaaacgacggccagt |  |
| P5 | atggcattagaaatagatatag |  |
| P6 | atttgatttaatttttaaggtgcgc |  |
| P7 | ctcgctctgctaatcctgttac |  |
