# Supplementary figures and images for: UIS2: A Unique Phosphatase Required for the Development of Plasmodium Liver Stages
Source: PLoS Pathog. 2016 Jan 6;12(1):e1005370. doi: 10.1371/journal.ppat.1005370 (PMC4712141; doi:10.1371/journal.ppat.1005370)

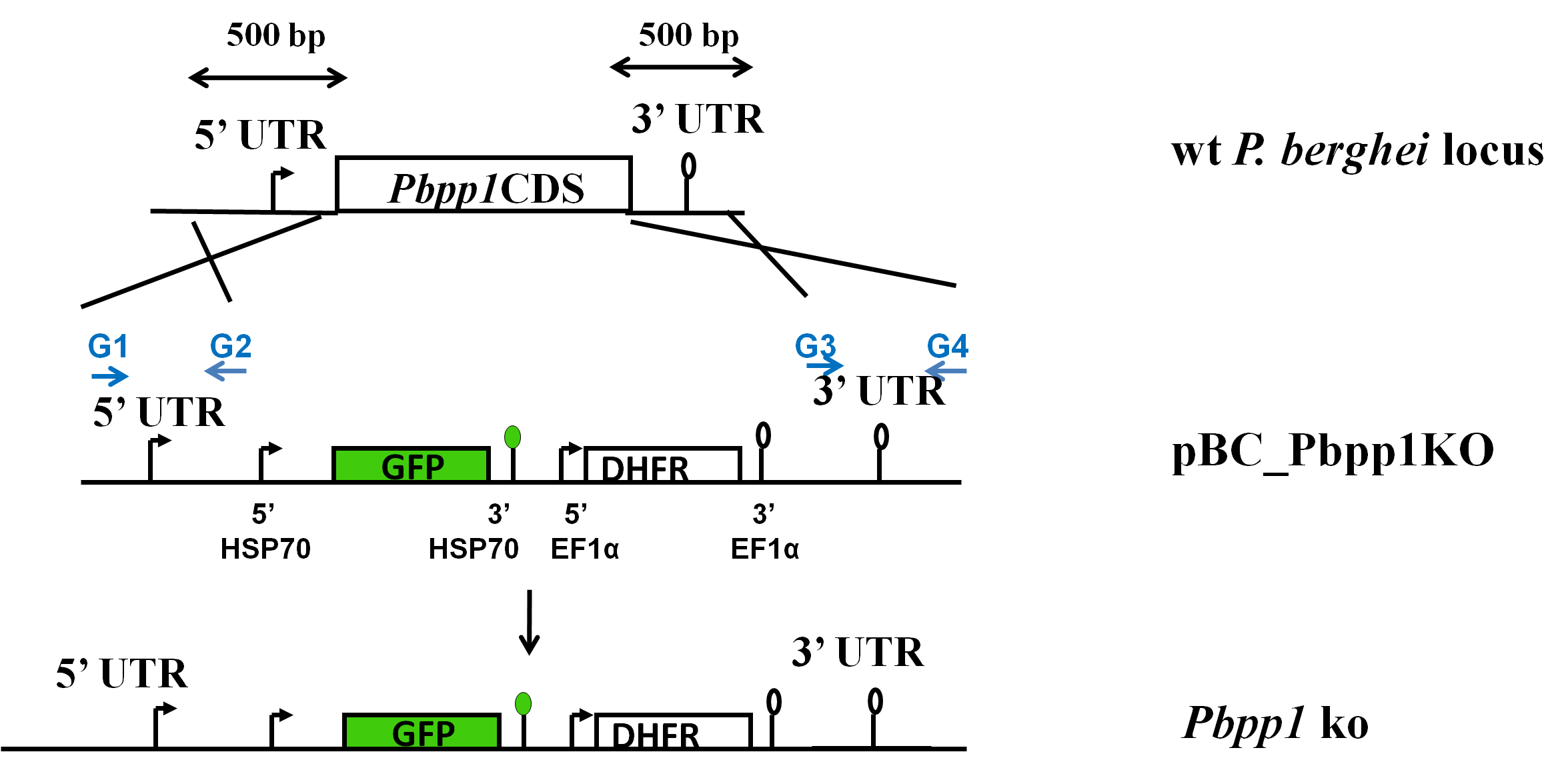

Supplement: S1 Fig — A double cross-over knockout strategy was used to knockout Pbpp1. hDHFR, human dihydrofolate reductase. Parasites were found in the blood smear of mice 24 hours post-transfection of the linearized construct. The mice were then treated with pyrimethamine. No parasites were observed 14 days post pyrimethamine treatment. Two attempts to knockout Pbpp1 failed. Related to Fig 1. (TIF) [file ppat.1005370.s003.tif]

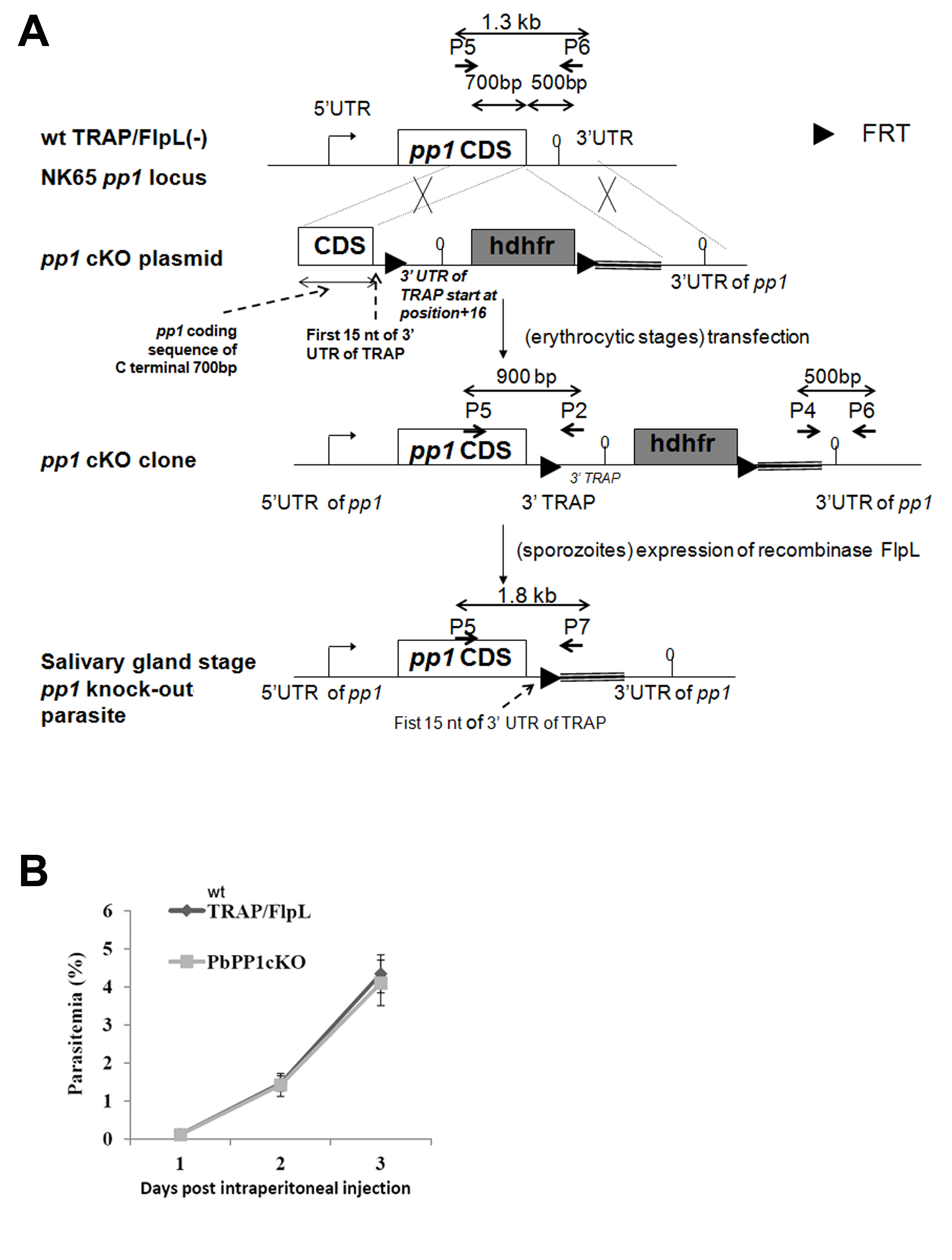

Supplement: S2 Fig — (A) The targeting plasmid for generating the Pbpp1 cKO contains the 3’ end (700 bp) of the pp1 coding sequence (box CDS), a fragment including 0.6 kb of TRAP 3’ regulatory sequence (lollipop) and the hdhfr marker cassette (gray box), the plasmid backbone (thick line), and pp1 3’ regulatory sequence (500 bp, lollipop). The linearized plasmid was integrated at the cognate locus into wt TRAP/FlpL(-) NK65 parasite via double crossover recombination, generating the pp1 cKO clone. (B) Pbpp1 expression cassette is intact in Pbpp1 cKO blood stage. Swiss Webster mice (5 per group) were injected intra-peritoneally with 200 μl of blood infected with Pbpp1cKO or wt TRAP/FlpL(-) parasites (1% parasitemia). The parasitemia of the recipient mice was checked in Giemsa-stained blood smears. Since the FlpL recombinase is expressed in midgut sporozoites, Pbpp1 is only disrupted in Ssp. Related to Fig 1. (TIF) [file ppat.1005370.s004.tif]

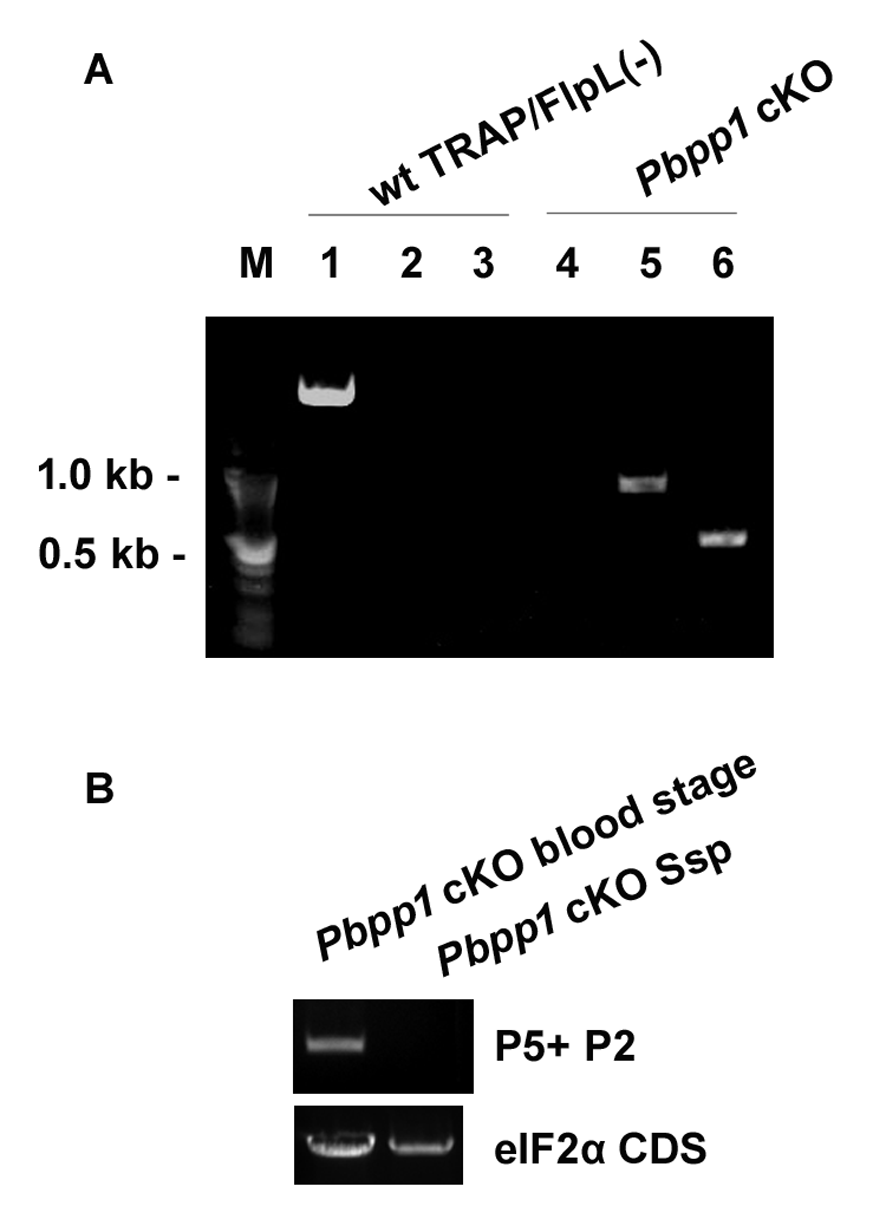

Supplement: S3 Fig — The primers used for integration-specific PCR analysis are indicated as arrows in S2 Fig. (A) Integration-specific PCR analysis of the uis2 loci of the wt TRAP/FlpL (-) NK65 and uis2 cKO blood stage clones. Lanes 1 and 4: primers P5+P6; lanes 2 and 5: primer P5+P2; lanes 3 and 6: primer P4+P6. (B) Pbpp1 locus is disrupted in Pbpp1 cKO Ssp. PCR amplification from gDNA of intact uis2 cKO blood stage parasites or from pp1 cKO Ssp using primers P5+P2 were performed. Control depicts PCR amplification of the eIF2α coding sequence. Related to Fig 1. (TIF) [file ppat.1005370.s005.tif]

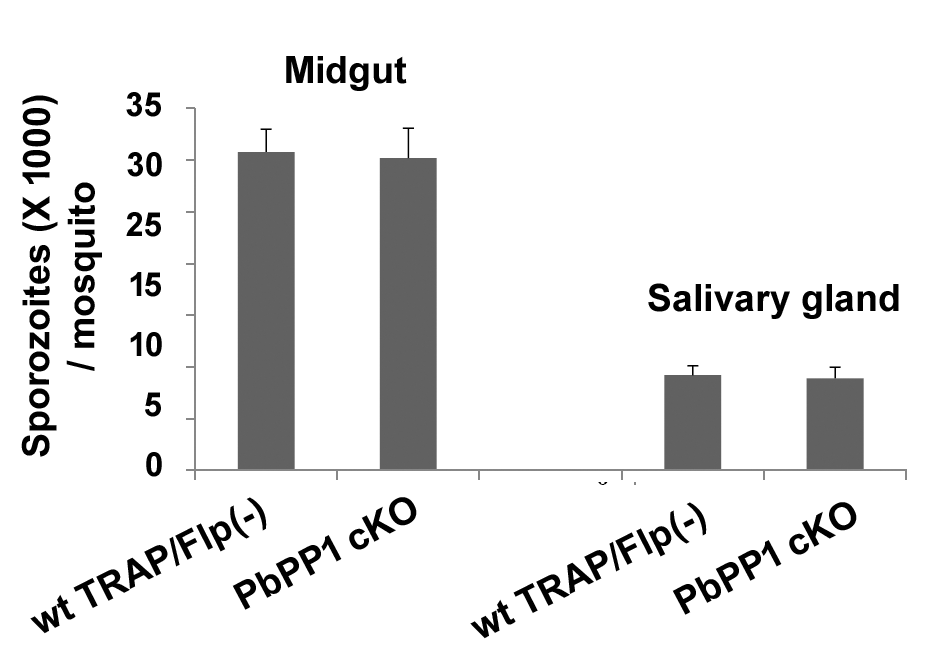

Supplement: S4 Fig — Sporozoite numbers were counted in three different mosquito cycles. Related to Fig 1 (TIF) [file ppat.1005370.s006.tif]

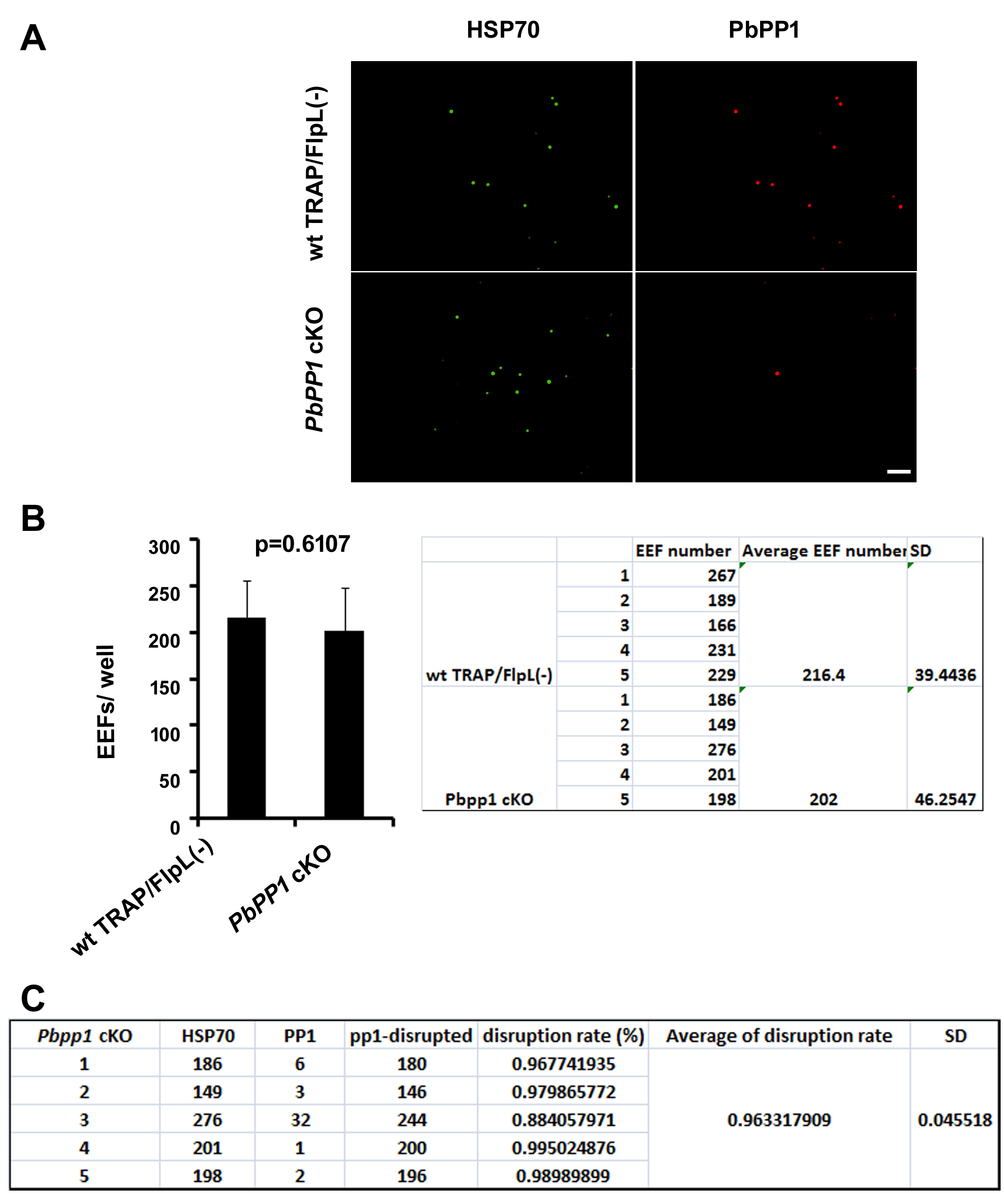

Supplement: S5 Fig — (A) Twenty thousand wild type TRAP/FlpL(-) or Pbpp1 cKO sporozoites were added to 1x10^5^ HepG2 cells. Fourty-eight hours post infection hepatic parasites were stained with anti-PbHSP70 and anti-PbPP1. Bar, 100 μm. (B) The HSP70 stained liver stage parasites were counted. The EEF (Liver stage) numbers were indistinguishable between wild type TRAP/FlpL(-) and Pbpp1 cKO parasites. P value was calculated by t test. (C) The HSP70 stained and PP1 stained liver stage parasites were counted in Pbpp1 cKO infected HepG2 cells. The Pbpp1 disruption rate was calculated from 5 independent experiments. Related to Fig 1. (TIF) [file ppat.1005370.s007.tif]

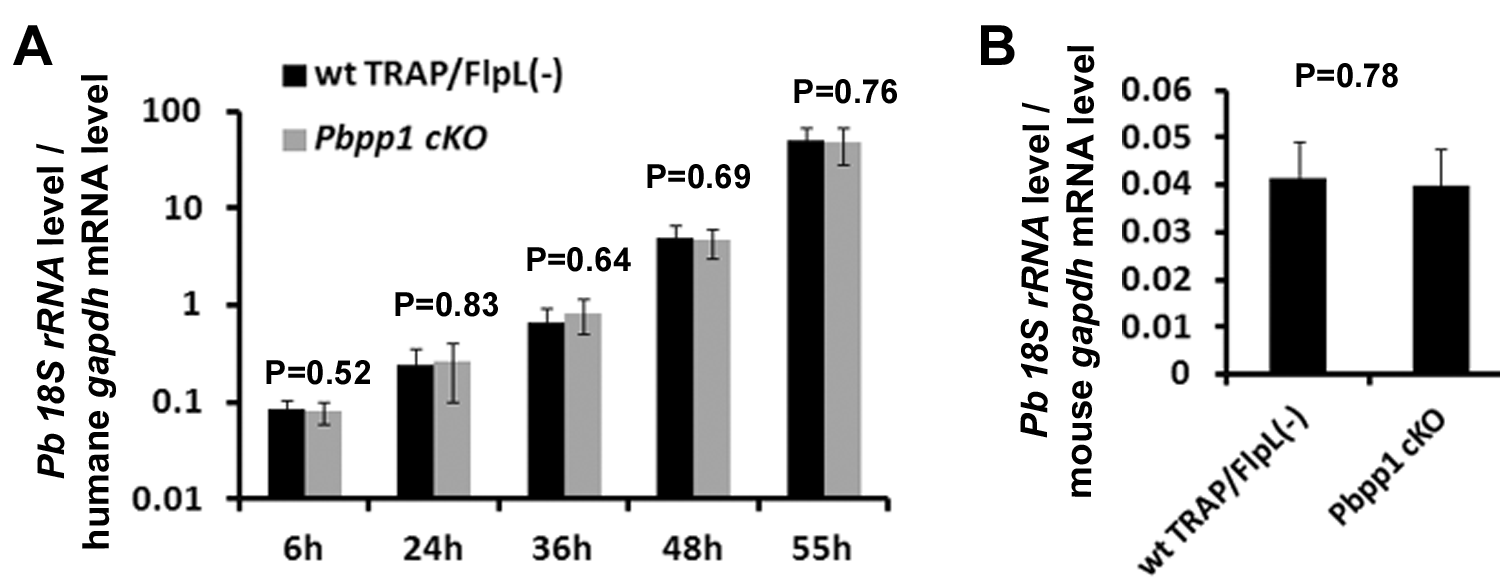

Supplement: S6 Fig — (A) Twenty thousand wild type TRAP/FlpL(-) or Pbpp1 cKO sporozoites were added to 1x10^5^ HepG2 cells and grown for 6, 24, 36, 48, and 55 h. P berghei 18S rRNA copy number was measured by qPCR. Human gapdh was used as internal control. There was no significant difference between wt and Pbpp1 cKO. (B) C57BL/6 mice (6 weeks old, five mice per group) were intravenously injected with 1x10^4^ wt TRAP/FlpL(-) or Pbpp1cKO sporozoites. Liver-stage parasite burden was measured 42 hours post infection by qPCR, and shown are the mean ± SD. Pbpp1 cKO Ssp developed normally in mice. P value was calculated by t test. Related to Fig 1. (TIF) [file ppat.1005370.s008.tif]

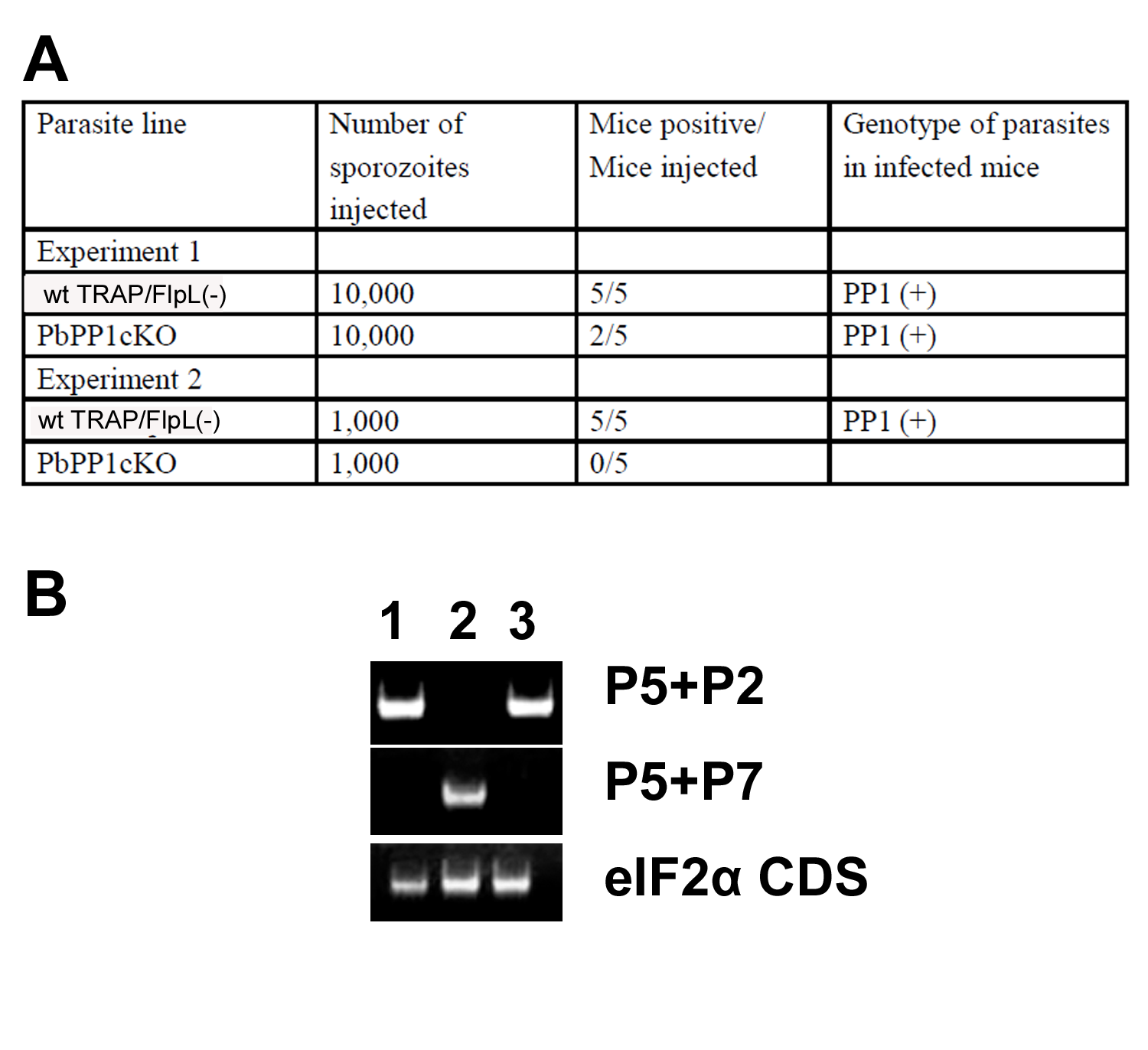

Supplement: S7 Fig — (A) Infectivity of wt TRAP/FlpL(-) and Pbpp1 cKO sporozoites after intravenous injection of C57BL/6 mice. Genotype of blood stage parasites was analyzed from the Pbpp1 cKO sporozoites infected mice. The parasites were pp1 (+); therefore, the pp1 locus had not been disrupted by the recombinase FlpL. The incomplete excision in the FlpL/FRT-mediated conditional mutagenesis system was previously reported by Combe et al. [14]. (B) Genotype of blood stage parasites from the Pbpp1cKO sporozoites infected mouse performed by gDNA PCR. Lane 1, intact Pbpp1 cKO blood stage parasites; lane 2, Pbpp1 cKO Ssp; lane 3, blood stage parasites from a Pbpp1 cKO sporozoites infected mouse. Primers P5+P7 (S2 Fig) were used to verify the disruption of Pbpp1 3’UTR. Primers P5 and P2 were used to verify the intact Pbpp1 locus. Related to Fig 1. (TIF) [file ppat.1005370.s009.tif]

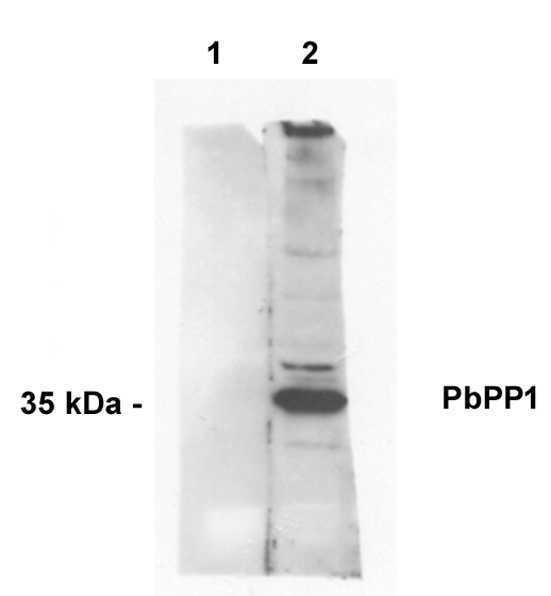

Supplement: S8 Fig — The proteins from 5X105 P. berghei blood stage parasites were separated by SDS-PAGE followed by Western blot using naive mouse serum (Lane 1) and mouse anti-PbPP1 antibody (Lane 2), respectively. The mouse anti-PbPP1 antibody recognized the 35 kDa endogenous PP1 from the lysates of P. berghei blood stage parasites. Related to Fig 2. (TIF) [file ppat.1005370.s010.tif]

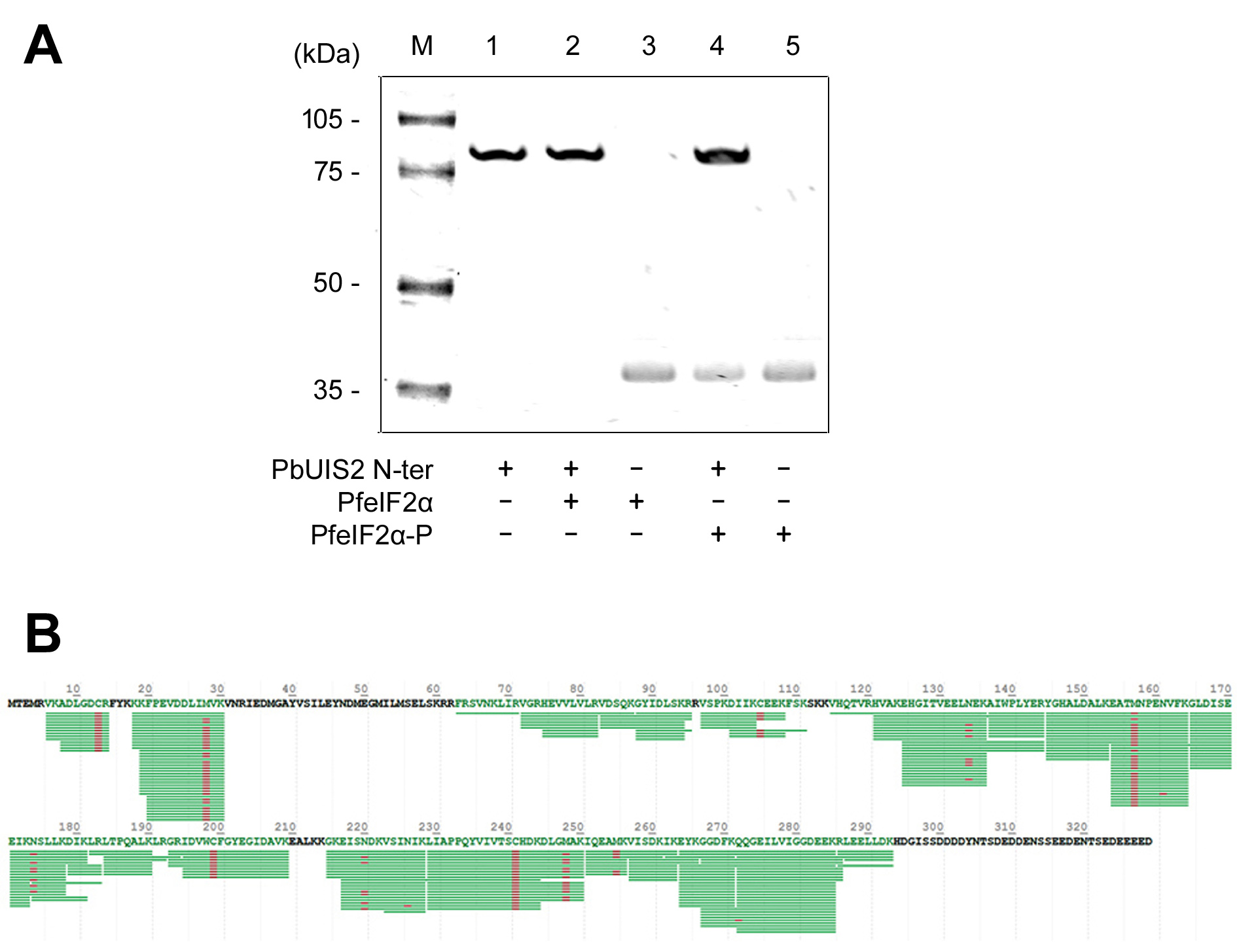

Supplement: S9 Fig — (A) The N-terminus of PbUIS2 pulled down PfeIF2α-P. The PbUIS2 N-ter was fused to GST-tag at its N-terminus and His-tag at its C-terminus. After 2-step affinity purification, the E. coli expressed fusion protein (85 kDa) was immobilized on glutathione sepharose 4B. After incubation with purified recombinant PfeIF2α or PfeIF2α-P, the sepharose was washed three times with high-salt NETN buffer (300 mM NaCl, 20 mM Tris-HCl, pH 8.0, 0.5 mM EDTA, and 0.5% (v/v) Nonidet P-40). The retained proteins were detected by SDS-PAGE followed by coomassie brilliant blue staining. Lane 1: PbUIS2 N-ter. Lane 2: proteins retained on the glutathione sepharose 4B after the pull down assays with non-phosphorylated PfeIF2α. Lane 3: non-phosphorylated PfeIF2α input control. Lane 4: proteins retained on the glutathione sepharose 4B after the pull down assays with PfeIF2α-P. Lane 5, PfeIF2α-P control. (B) Mass spectrometry shows that the protein bound to the PbUIS2 N-ter is indeed PfeIF2α-P. The PbUIS2 N-ter pulled down protein was analyzed by mass spectrometry. Peptides denoted in green are peptides identified in our analysis. Each green line under a peptide denotes the number of times the peptide has been identified in this analysis (spectral counts). A red line below a peptide indicates that the corresponding amino acid carries a modification (in vivo or in vitro). The modification shown here are: C = Carbamidomethylation (+57 for alkylation of cysteines, that is a result of the sample preparation); M = oxidation (+16 for oxygen addition, common in gel digestion); Q, N = deamidation (+1, usually an in vitro modification enhanced by storing samples at high pH. Sample digestion was performed at pH = 8 overnight). Related to Fig 2. (TIF) [file ppat.1005370.s011.tif]

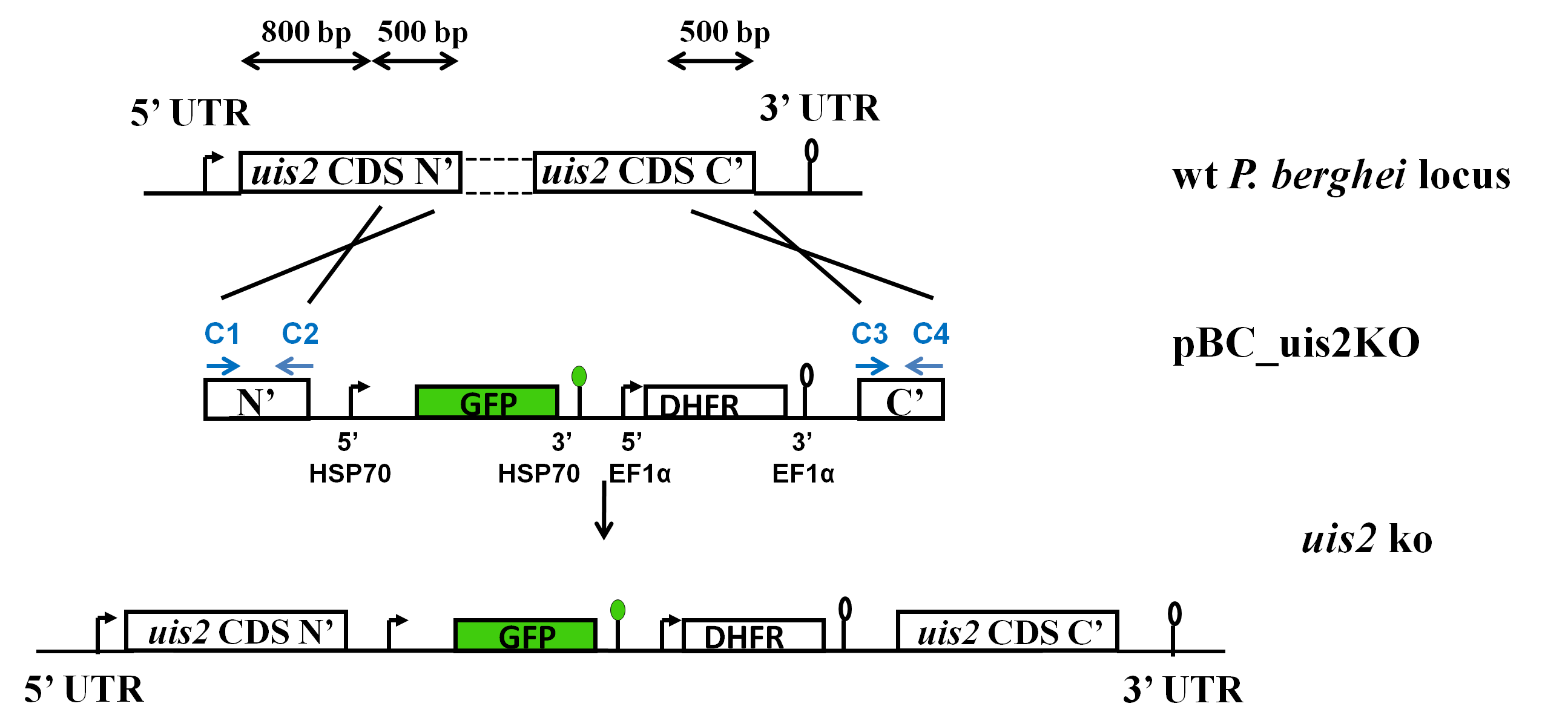

Supplement: S10 Fig — A double cross-over knockout strategy used to attempt a knockout uis2 in P. berghei. The uis2 KO plasmid pBC_uis2KO contains a 500-bp PCR fragment from uis2 coding sequence (CDS, 800–1300 bp), GFP cassette, DHFR cassette, and a 500 bp PCR fragment from the uis2 C terminal CDS. DHFR, dihydrofolate reductase. Three independent attempts to generate Pbuis2 knock-out parasite failed. (TIF) [file ppat.1005370.s012.tif]

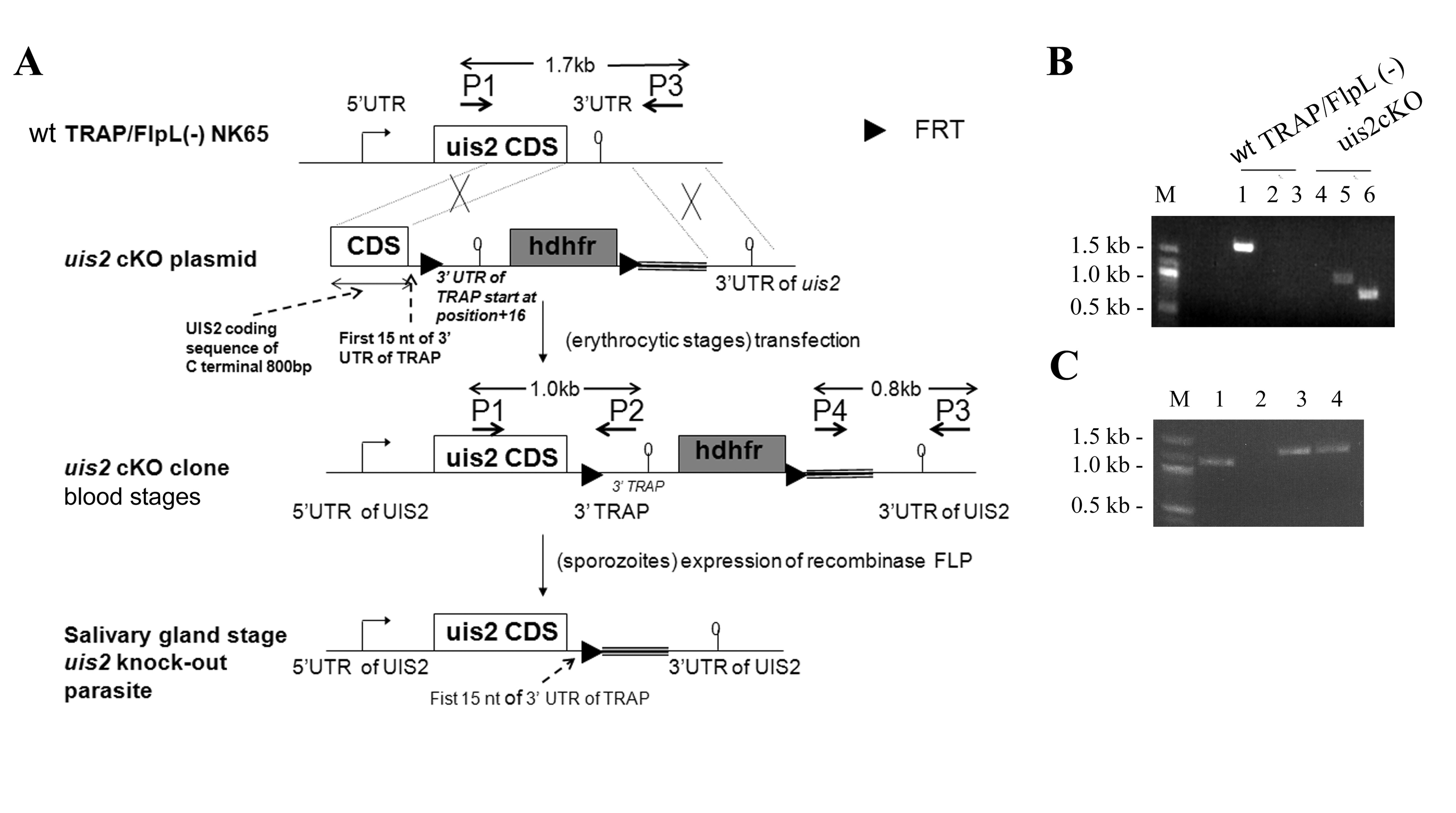

Supplement: S11 Fig — (A) Schematic representations of the uis2 locus in the wild type TRAP/FlpL(-) NK65 clone and in the uis2 cKO clone. The uis2 cKO plasmid contains the 3’ end (800 bp) of the uis2 coding sequence (box CDS), a fragment including 0.6 kb of TRAP 3’ regulatory sequence (lollipop) and the hdhfr marker cassette (gray box), the plasmid backbone (thick line), and uis2 3’ regulatory sequence (770 bp, lollipop). The linearized plasmid integrated at the cognate locus into wt TRAP/FlpL(-) NK65 parasite via double crossover recombination, generating the uis2 cKO clone. The primers used for integration-specific PCR analysis are indicated as arrows. (B) Integration-specific PCR analysis of the uis2 loci of the wt TRAP/FlpL (-) NK65 and uis2 cKO clones. Lanes 1 and 4: primers P1+P3; lanes 2 and 5: primer P1+P2; lanes 3 and 6: primer P3+P4. (C) Pbuis2 locus is disrupted in Pbuis2 cKO Ssp. PCR amplification from gDNA of intact uis2 cKO blood stage parasites (lane 1) or uis2 cKO Ssp (lane 2) using Primers P1+P2. Controls are shown in lanes 3 and 4: PCR amplification of eIF2α coding sequence from uis2 cKO blood stage parasites (lane 3) and from uis2 cKO Ssp (lane 4). Related to Fig 4. (TIF) [file ppat.1005370.s013.tif]

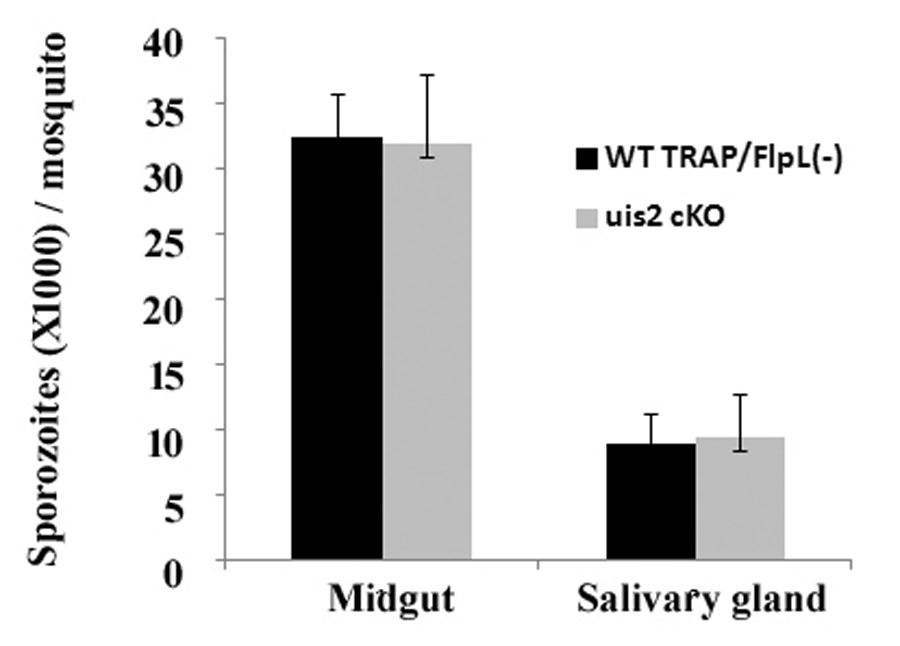

Supplement: S12 Fig — Sporozoite numbers were counted in three different mosquito cycles. Related to Fig 4. (TIF) [file ppat.1005370.s014.tif]

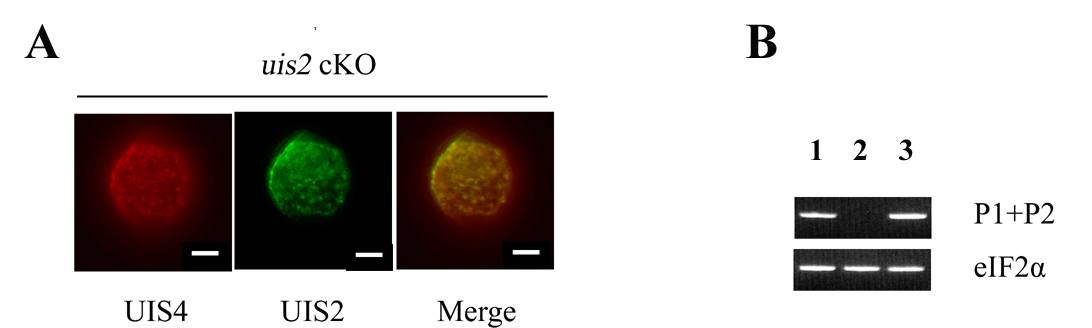

Supplement: S13 Fig — (A) A few uis2 (+) parasites still remained from the liver stages originated from uis2 cKO Ssp. The figure represented one of them stained with anti-UIS2 and anti-UIS4 sera. Bars, 10 μm. (B) The genotype of the blood stage parasites originated by uis2 cKO sporozoites was uis2 (+). gDNA PCR amplifications were performed using primers P1 and P2 (S11 Fig) and uis2 expression cassette from intact uis2 cKO blood stage parasites (lane 1), uis2 cKO Ssp (lane 2), and the erythrocytic stages parasites from a mouse infected with uis2 cKO Ssp (lane 3). eIF2α was used as internal control. Related to Table 1. (TIF) [file ppat.1005370.s015.tif]
